# Altered Effective Connectivity within an Oculomotor Control Network in Unaffected Relatives of Individuals with Schizophrenia

**DOI:** 10.3390/brainsci11091228

**Published:** 2021-09-17

**Authors:** Matthew Lehet, Ivy F. Tso, Sohee Park, Sebastiaan F. W. Neggers, Ilse A. Thompson, Rene S. Kahn, Katharine N. Thakkar

**Affiliations:** 1 Department of Psychology, Michigan State University, East Lansing, MI 48824, USA; lehetmat@msu.edu; 2 Department of Psychiatry, University of Michigan, Ann Arbor, MI 48109, USA; ivytso@med.umich.edu; 3 Department of Psychology, Vanderbilt University, Nashville, TN 37235, USA; sohee.park@vanderbilt.edu; 4 Department of Psychiatry, University Medical Center Utrecht, 3584 CX Utrecht, The Netherlands; B.Neggers@umcutrecht.nl (S.F.W.N.); ilsethompson@hotmail.com (I.A.T.); 5 Department of Psychiatry, Icahn School of Medicine at Mount Sinai, New York, NY 10029, USA; ene.kahn@mssm.edu; 6 Department of Psychiatry and Biobehavioral Medicine, Michigan State University, East Lansing, MI 48824, USA

**Keywords:** schizophrenia, endophenotype, response inhibition, effective connectivity

## Abstract

The ability to rapidly stop or change a planned action is a critical cognitive process that is impaired in schizophrenia. The current study aimed to examine whether this impairment reflects familial vulnerability to schizophrenia across two experiments comparing unaffected first-degree relatives to healthy controls. First, we examined performance on a saccadic stop-signal task that required rapid inhibition of an eye movement. Then, in a different sample, we investigated behavioral and neural responses (using fMRI) during a stop-signal task variant that required rapid modification of a prepared eye movement. Here, we examined differences between relatives and healthy controls in terms of activation and effective connectivity within an oculomotor control network during task performance. Like individuals with schizophrenia, the unaffected relatives showed behavioral evidence for more inefficient inhibitory processes. Unlike previous findings in individuals with schizophrenia, however, the relatives showed evidence for a compensatory waiting strategy. Behavioral differences were accompanied by more activation among the relatives in task-relevant regions across conditions and group differences in effective connectivity across the task that were modulated differently by the instruction to exert control over a planned saccade. Effective connectivity parameters were related to behavioral measures of inhibition efficiency. The results suggest that individuals at familial risk for schizophrenia were engaging an oculomotor control network differently than controls and in a way that compromises inhibition efficiency.

## 1. Introduction

The ability to respond flexibly in the context of ever-changing internal goals and environmental demands is a critical cognitive function. Central to such flexibility is inhibitory control: the ability to suppress contextually irrelevant or inappropriate actions. Two broad forms of inhibitory control were described: proactive and reactive [1,2]. Proactive inhibition, or action restraint, refers to the preparation to withhold a response. Reactive inhibition, or action cancellation, refers to the outright stopping of an initiated movement plan on the basis of an external cue. Both proactive and reactive inhibition impairments were associated with a range of neuropsychiatric conditions [3], including schizophrenia [4,5,6,7,8,9,10,11], which is a neurodevelopmental disorder with high heritability that is characterized by hallucinations and delusions, low motivation, diminished expressivity, disorganized thought, and broad cognitive deficits [12,13]. In individuals that are diagnosed with schizophrenia, poor inhibitory control is one such cognitive deficit (see [14] for others) that was linked with poor psychosocial outcomes [15,16,17,18,19], highlighting its clinical relevance. Whether inhibitory control deficits are proximal illness mechanisms or reflect familial vulnerability was addressed in studies of unaffected first-degree relatives of individuals with schizophrenia.

One measure of proactive inhibitory control that was extensively studied in unaffected first-degree relatives of individuals with schizophrenia is performance on the antisaccade task [20,21,22,23,24]. The antisaccade task requires participants to make a saccade in the opposite direction of a visual target [25]. Individuals with schizophrenia have higher error rates compared to healthy controls (reviewed in [20,26]) and this deficit appears to be stable across illness episodes [27]. Unaffected first-degree relatives also show greater error rates than healthy controls [27,28,29,30,31,32,33,34], but this finding was not consistently reported [24,35,36,37,38,39,40,41,42,43,44,45]. Differing inclusion criteria for unaffected relatives is one source of variability across studies: antisaccade deficits in non-psychotic relatives are more prominent in those studies that did not exclude a history of other Axis 1 diagnoses [46].

Reactive inhibitory control has been less researched in unaffected relatives. Performance on the so-called stop-signal task is considered the gold-standard metric of reactive inhibition. The stop-signal task involves speeded responses to a signal that cues a movement (go trial). However, on a subset of trials, a second signal appears after a short delay, which instructs the participant to stop or alter their planned response (stop trial). Performance on stop-signal tasks can be modeled as a race between competing processes (GO and STOP). This modeling permits calculation of how long the STOP process takes to complete (i.e., the speed of inhibition), namely, the stop-signal reaction time (SSRT) [47]. While the SSRT reflects reactive inhibitory speed, stop-signal tasks also involve proactive inhibition: compared to a speeded reaction time (RT) task that does not require stopping, participants slow their responses when a stop-signal might occur. As stop-signal likelihood increases, GO RTs increase parametrically [48,49,50]. That is, participants prepare to stop. Directly comparing speeded reaction time tasks to no stop-signal trials in stop-signal tasks reveals a context effect whereby the possible occurrence of the stop-signal reduces movement times in addition to increasing reaction times. This approach to quantifying proactive inhibition has proved fruitful in a variety of clinical populations [51,52,53,54,55,56]. Reactive inhibition in the context of the stop-signal was widely studied among individuals with schizophrenia, with slowed SSRT extensively reported in both manual [5,19,57,58,59,60,61,62,63,64,65,66] and saccadic [10,67,68,69] versions of the task; however, see [9,70,71,72,73].

Only a handful of studies have investigated performance on the stop-signal task in unaffected relatives of individuals with schizophrenia. In these studies, relatives did not generally show reliable slowing of SSRT compared to healthy controls [5,9,60,66,70,71,74], although the largest study to date observed longer SSRTs in relatives of individuals with schizoaffective disorder bipolar type than healthy controls [5]. To date, all stop-signal studies conducted in healthy relatives have used keypress versions of the task. However, there are several reasons to investigate reactive inhibition in the eye movement domain, using tasks where participants must inhibit or redirect a prepared saccade. First, there is evidence for separable central and peripheral inhibitory mechanisms [75], which differ in the extent to which they are effective at stopping eye versus manual movements. Thus, intact reactive inhibition of manual movements does not imply intact inhibition of eye movements. Second, a notable advantage to using saccadic versions of the stop-signal task is the robust body of neurophysiology work in non-human primates characterizing specific neuronal populations involved in canceling or changing a saccade using tasks akin to those used with human participants. This animal work can thus inform findings in clinical populations.

A network of cortical and subcortical regions that were identified using non-human neurophysiology play a role in executing saccades, exerting inhibitory control over gaze, or both. The frontal eye fields (FEF) and superior colliculus both contain populations of fixation and movement neurons that are active during fixation and saccade preparation, respectively [76,77,78]. During the stop-signal task, stop-signal presentation is followed by rapid attenuation of movement neuron activity [79,80,81], whereas activity in fixation neurons increases [82,83]. Modulation of movement neuron activity can transpire via interactions with fixation neurons. Alternatively, output from the basal ganglia can directly inhibit the superior colliculus and inhibit movement neurons in the FEF by way of the thalamus, which has the net effect of inhibiting saccade production [84,85,86].

Neurons in the medial frontal cortex are also engaged during stop-signal task performance, but the pattern of modulation is different than in the FEF and superior colliculus. The medial frontal cortex includes pre-supplementary and supplementary motor areas, as well as the supplementary eye fields (SEF), which together comprise the supplementary motor complex. In non-human primates, SEF activity changes following stop-signal onset, but this modulation likely reflects later evaluative processes [87,88,89] rather than directly reflecting inhibitory mechanisms. The SEF are poised to regulate saccade likelihood by facilitating either fixation or saccade execution [87,90,91]. In contrast to data from non-human primates, however, human functional MRI studies suggest that the supplementary motor complex may *directly* implement reactive inhibition, possibly by influencing motor effectors through the basal ganglia [92,93].

Another region that facilitates top-down inhibitory control is the right inferior frontal cortex (rIFC), which was suggested to play a role in detecting and responding to cues to alter or inhibit planned actions [94,95,96,97,98], including eye movements [82,99]. This inhibition is thought to occur through coordination with the SEF, but also by way of connections to the sub-thalamic nucleus of the basal ganglia [100,101,102,103,104].

Thus, the SEF, FEF, rIFC, caudate, superior colliculus, and thalamus make up a putative saccadic control circuit, which is further supported by anatomical between-region connections. The FEF and SEF are reciprocally connected [105,106,107], with descending connections to the superior colliculus [108,109,110], striatum [105,107,111,112,113], and thalamus [105,107,113,114]. Connections from the striatum and the superior colliculus to cortical oculomotor regions (i.e., the FEF and SEF) pass through the thalamus [115,116,117] and are themselves reciprocally connected [84,118,119,120]. Finally, the IFC projects to the caudate [112], and is bidirectionally connected with the SEF [107,121] and thalamus [122], and may thus exert control over saccade plans. This network, which is supported by both physiology and anatomical connections, allows for proactive and reactive control over saccade generation.

Prior investigations that assessed neural responses during inhibitory control in unaffected relatives of individuals with schizophrenia focused on functional responses within modular regions. Two fMRI studies using the antisaccade paradigm found differences between relatives and controls in the SEF/anterior cingulate, FEF, and caudate, suggesting functional differences within the oculomotor network described above [123,124]. The neural basis of inhibitory control in unaffected relatives was also examined in manual stop-signal tasks [9,71,74,125]. Three of these studies [9,74,125] reported reduced striatal modulation as a function of stop-signal probability in unaffected relatives compared to healthy controls, indicating altered proactive inhibition processes. Deficits associated with proactive inhibition among siblings are further indicated by differences in EEG P300 potentials that may reflect reduced readiness or less proactive inhibition [60]. In contrast, patterns of brain activation associated with reactive inhibition appear to be largely unaffected among siblings of individuals with schizophrenia, at least in manual stop-signal tasks [9]. These prior results suggest differences between relatives and healthy controls in how inhibitory control, especially proactive inhibitory control, is engaged within the motor control network. However, prior stop-signal task studies in unaffected relatives focused on manual versions of the task and did not explore the network dynamics underlying task performance that are likely important for understanding the mechanisms of putative behavioral impairments.

Here, we report data from two studies comparing healthy controls to unaffected relatives of persons with schizophrenia in two variants of the saccadic stop-signal task. For the first study, we report behavioral results from twelve unaffected relatives (REL) and fourteen healthy controls performing a saccadic stop-signal task. For the second study, we report behavioral and neuroimaging results from a different group of twenty-two unaffected siblings (SIB) and twenty-three healthy controls in an fMRI study using a saccadic stop-signal task variant, namely, the search-step task [126,127]. We examine neural responses using both a regions-of-interest analysis and a dynamic causal modeling (DCM) approach. The DCM framework [128,129,130] allowed for the interrogation of causal connections between regions and identifies how the need to exert inhibitory control modulated these causal relationships between regions. Our findings speak to the degree to which inhibitory control impairments and the accompanying abnormalities in functional brain activity are associated with familial risk for schizophrenia.

## 2. Materials and Methods

### 2.1. Experiment 1

In this experiment, we examined performance on a saccadic stop-signal task in a small sample of unaffected first-degree relatives of persons with schizophrenia and healthy controls. We compared the SSRT (STOP latency) and the speed of executing a saccade (GO latency) across groups.

#### 2.1.1. Participants

Twelve unmedicated and unaffected first-degree relatives (REL) were recruited from a registry of individuals with schizophrenia that had participated in previous studies, as well as through a mental health advocacy organization. Structured clinical interviews (SCID-IV [131]) were used to verify proband diagnosis whenever possible (proband diagnoses for six REL were confirmed). Fourteen demographically matched healthy control participants (HC) were selected from a larger sample of healthy controls that participated in this study (reported in [10,67]), which were recruited via community advertisements. Both HC and REL were unmedicated and without a personal history of Diagnostic and Statistical Manual of Mental Disorders, fourth edition (DSM-IV) Axis I disorders, as determined using SCID-IV [131]. All participants were screened and were excluded if they were found positive for neurological disorders, substance use within the past 6 months, inability to fixate, history of head injury, and excessive sleepiness. HC were additionally excluded if they had a self-reported family history of a psychotic disorder. All participants had normal or corrected-to-normal vision. The Adult North American Reading Test (ANART [132,133]) or Wechsler Abbreviated Scale of Intelligence [134] was used to assess IQ. Handedness was assessed with the Modified Edinburgh Handedness Inventory [135]. Controls and healthy relatives were matched regarding age, sex, education, IQ, and handedness (see Table 1). All participants were paid for their time and gave written informed consent approved by the Vanderbilt Institutional Review Board.

#### 2.1.2. Apparatus and Stimuli

An EyeLink II eyetracker (SR Research, Canada) was used to assess eye positions. The sampling rate was set to 250 Hz with an average gaze position error <0.5° and noise limited to <0.01° RMS. Saccades were detected online using a velocity criterion (35°/s. Each participant was seated with their head in a chin rest positioned 57 cm from the display. The fixation and targets subtended 1° and were light gray (34 cd/m^2^) on a darker gray (18 cd/m^2^) background.

#### 2.1.3. Stop-Signal Task

**Figure 1:**
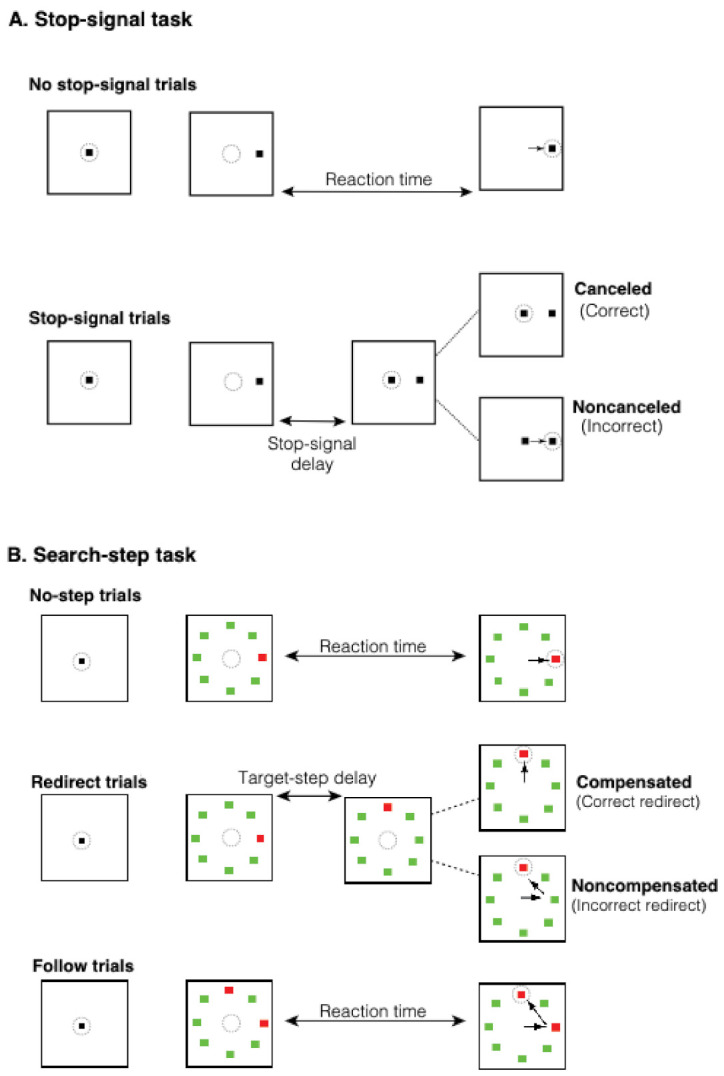
Experimental tasks. Dotted circles indicate gaze position and the arrow indicates the direction of the saccade. (**A**) The saccadic stop-signal task. The task comprised two randomly interleaved trial types: no stop-signal and stop-signal trials. Participants began each trial by maintaining their gaze on a central fixation point. Following a variable fixation period, the fixation point disappeared and simultaneously reappeared to the left or right of the central fixation. Participants were instructed to look at the peripheral target as quickly as possible. In stop-signal trials, however, the central fixation target reappeared after a short, variable delay (stop-signal delay (SSD)). In these trials, participants were instructed to withhold the planned saccade to the peripheral target. Trials in which the participant was successful in withholding the planned saccade were termed canceled trials, and trials in which the participant erroneously looked at the peripheral target were termed noncanceled trials. (**B**) The search-step task. Three randomly interleaved trial types comprised the task: *no-step, redirect, and follow*. Participants began every trial by maintaining their gaze on a central fixation point. Following a variable fixation period, the fixation point disappeared and an eight-element array appeared. In no-step and redirect trials, the search array contained one red target and seven green distractors (T1). In no-step trials, this array was visible for the duration of the trial. In redirect trials, the red target changed location (T2) through an isoluminant color change occurring with a varying delay after the initial array (target-step delay (TSD)). In follow trials, the initial array contained two red targets that were visible for the duration of the trial. In no-step and redirect trials, participants were asked to make a saccade to T1 as quickly as possible. If the target changed locations (redirect trials), participants were to inhibit the saccade to T1 and instead saccade to T2 as quickly as possible. In follow trials, participants were asked to make saccades to each red target (the order did not matter). Redirect trials where participants made a saccade directly to T2 were termed compensated trials. Redirect trials where participants made their first saccade to T1 were termed noncompensated trials; noncompensated trials frequently involved a corrective saccade to the new target location.

Participants performed a saccadic stop-signal task (Figure 1A). The task consisted of two trial types: *no stop-signal* (70% of trials) and *stop-signal* (30% of trials) that were randomly interleaved. Each trial began with a central fixation that varied in duration between 500–1000 ms. After the fixation disappeared, a peripheral target appeared at one of two randomly selected locations (left or right) equidistant (8.5°) from the central fixation. Participants were instructed to look directly at the target as quickly as possible. On stop-signal trials, after a variable delay (*stop-signal delay* (SSD)) following target onset, the fixation was re-illuminated, cueing participants to inhibit the planned saccade to the target. Stop-signal trials were termed *canceled* or *noncanceled* depending on whether participants inhibited or did not inhibit the saccade, respectively.

Inhibiting responses become increasingly difficult as the SSDs increase. SSDs were dynamically adjusted using a 1-up/1-down tracking procedure, aiming for successful inhibition in 50% of the stop-signal trials [136]. The initial SSD was 225 ms and either increased or decreased by 47 ms when the participant succeeded or failed to inhibit, respectively. The testing session consisted of a practice block of 60 trials and 4 experimental blocks of 120 trials each.

Participants were instructed that the speed of the saccade was equally as important as inhibiting the saccade on stop-signal trials in order to deter participants from waiting for the fixation to reappear. They were also told that inhibiting the saccade on stop-signal trials would not always be possible.

#### 2.1.4. Task Performance

Behavioral performance was evaluated through measurements of SSD and saccadic RT in no stop-signal and stop-signal trials. Performance in the stop-signal task can be calculated based on a mathematical model that assumes a race between independent processes that generate (GO process) and inhibit (STOP process) the movement [47]. The response is executed if the GO process finishes before the STOP process and inhibited if the STOP process finishes first. The latency of the GO process can be measured directly from no stop-signal RTs but the latency of the STOP process must be estimated. The independent race model provides an estimate of the time needed to respond to the stop-signal and cancel the movement, referred to as the *stop-signal reaction time* (SSRT). The SSRT was calculated using the integration method [47,137,138], which is the most reliable and least biased method for estimating the SSRT in stop-signal tasks that utilize a dynamic tracking procedure [139]. To compute the SSRT using the integration method, each participant’s no-step trial RTs were sorted in ascending order. Then, the RT corresponding to the proportion of noncanceled trials was identified, and the mean SSD was subtracted from this RT.

#### 2.1.5. Statistical Analyses

Independent two-tailed t-tests were used to compare the SSRT and the proportion of canceled stop-signal trials between groups. The RT was examined using a mixed-model ANOVA, including trial (no stop-signal and noncanceled) as a within-participants variable and diagnostic group as a between-participants variable. Reported effect sizes for the ANOVAs are generalized eta squared.

### 2.2. Experiment 2

In this study, unaffected siblings of individuals with schizophrenia and healthy controls performed a variant of the saccadic stop-signal task, the search-step task, during fMRI. We compared the task performance, as well as both activation and effective connectivity, within a putative oculomotor control network between groups.

#### 2.2.1. Participants

Twenty-three siblings of persons with a confirmed schizophrenia spectrum disorder diagnosis (SIB) were recruited from a longitudinal study in the Netherlands (Genetic Risk and Outcome in Psychosis (GROUP) Investigators [140]). Note that the group in experiment 2 was all siblings as opposed to simply first-degree relatives, as was the case in experiment 1. In order to differentiate these two groups, the acronym SIB is used to refer to relatives in experiment 2 instead of REL, as was used in experiment 1. Diagnoses in SIB and schizophrenia spectrum disorder were established using Diagnostic and Statistical Manual of Mental Disorders, fourth edition (DSM-IV) criteria and verified with the Comprehensive Assessment of Symptoms and History interview [141] or Schedules for Clinical Assessment for Neuropsychiatry, version 2.1 [142]. Twenty-three healthy controls (HC) without a family or personal history of Axis I psychiatric diagnosis were selected from a larger group of HC participants that completed this study and that were recruited using community advertisements. This subsample of the larger control group was selected to be demographically matched based on gender and age to the SIB group. Participants in the SIB and HC groups were excluded if they had any current Axis I disorder (according to DSM-IV criteria). Participants were also excluded based on a history of neurological illness or head trauma, color blindness, or recent substance abuse. See Table 2 for the demographic information. The SIB and HC groups were matched for age, sex, IQ, handedness, and education. All participants gave written informed consent and were reimbursed for their participation. The study was approved by the Human Ethics Committee of the University Medical Center, Utrecht. Two siblings were excluded from the fMRI analyses: one due to motion and one due to acquisition artifacts, but were included in the behavioral results. One sibling showed an implausibly fast TSRT (less than 50 ms) and was excluded from all analyses. This resulted in 20 SIB included in the neuroimaging analyses and 22 included in the behavioral analyses.

#### 2.2.2. Search-Step Task

The saccadic search-step task (Figure 1B [126,127]) consisted of three trial types: *no-step* (30% of trials), *redirect* (40% of trials), and *follow* (30% of trials), which were randomly interleaved. Each trial had a 4 s duration with a fixation period that varied between 1000 and 2000 ms, which was followed by the presentation of an eight-element search array. On no-step and redirect trials, the search array had one red target and seven green distractors. The array elements were isoluminant, subtended 0.7° of visual angle, and were equidistant (9° of visual angle) from the screen center. On no-step trials, this array was presented for the duration of the trial. On redirect trials, the red target changed location through an isoluminant color change after a delay (the target-step delay (TSD)). On follow trials, the array appeared with two red targets and six green targets that stayed on screen for the duration of the trial. On no-step and redirect trials, participants were asked to saccade toward the red target (T1) as quickly as possible, and if the target jumped to a new location (redirect trials), they should inhibit the saccade to T1 and instead look at the new target (T2) as quickly as possible. On follow trials, participants were asked to look at the red targets, one after the other, in any order. Follow trials were not relevant to the research questions in this study, and therefore they are not discussed. To minimize the chance of saccades landing midway between T1 and T2, redirect and follow trial target locations were constrained such that there was at least 90 degrees between T1 and T2 [143].

Redirect trials where participants correctly inhibited their initial saccade and looked in the direction of T2 were termed *compensated* trials. Redirect trials where the participant incorrectly made their first saccade to T1 despite T2 appearing were termed *noncompensated* trials. Inhibition of the saccade to T1 becomes more difficult with longer duration TSDs [47,126]. The TSDs were adjusted dynamically with a one-up/one-down tracking procedure to achieve successful inhibition in roughly 50% of redirect trials. The initial TSD was 100 ms and then either increased or decreased by 67 ms after compensated or noncompensated redirect trials, respectively. For the sake of timing accuracy, TSDs were multiples of the screen refresh rate.

The experiment consisted of four runs that each lasted 5 min, with 60 trials in each run. Six 10 s rest blocks displaying only the fixation cross were interleaved as a baseline condition. Simulations were run before the experiment to identify a trial order that minimized correlations between the different model regressors in order to permit reliable estimations of the parameters. Participants were not explicitly instructed about the relative frequency of trial types. The experiment comprised 72 no-step trials, 72 follow trials, and 96 redirect trials. Participants were trained in the task before the scan. To reduce the chance of participants waiting for the target to move to a new location, we instructed them that speed on the no-step and follow trials was equally as important as successfully inhibiting a saccade to T1 on redirect trials and that it would not always be possible to inhibit the saccade to T1 on redirect trials.

#### 2.2.3. Apparatus and Stimuli

##### Stimulus Display

Stimuli were presented in the scanner using Presentation software (Neurobehavioral Systems) and displayed on an MR-compatible LED screen positioned in the rear of the bore that was viewed using a mirror attached to the head coil. Eye movements in the scanner were recorded at a sampling rate of 60 Hz with an MR-compatible infrared camera (Nordic Neuro Lab) using a video camera that was mounted on the head coil. Head-coil-mounted LEDs provided infrared illumination for eye tracking. ViewPoint eye-tracking software (Arrington Research) controlled the eye data acquisition. Stimulus timing was relayed by Presentation to the ViewPoint software and inserted into the eye movement recordings. The redirect trial accuracy was used to adaptively adjust the TSD. Eye position data across each trial were analyzed online, and the accuracy of the redirect trials was determined as follows. After every redirect trial, drift correction was applied to the eye position data using a mean eye position from the window 50 ms before and 50 ms after the array was presented. Trial accuracy was determined using a positional criterion. If the eye position exceeded 2° of visual angle from the fixation point after 100 ms and for more than two samples (33 ms), and the position was toward T2, then the trial was classified as correctly compensated. The TSD was increased on the next redirect trial. If the eye position was toward T1 then the trial was recorded as noncompensated, and the TSD was reduced on the following redirect trial. If the eye position was in the direction of neither T1 nor T2, the TSD was unchanged.

##### Eye Tracking Data Analysis

A semi-automated procedure in MATLAB (MathWorks) was used to conduct offline analysis of eye position data. The data were first differentiated to obtain a velocity signal and then filtered with a fifth-order Butterworth filter (40 Hz cutoff). Then, saccade onsets were automatically determined based on liberal velocity criteria. Saccades that were erroneously marked by this automated procedure, (e.g., due to camera noise and blinks) were manually removed. Saccade onset verification was done without knowledge of the experimental condition. Trials with saccade onset times <100 ms from the array onset were excluded from further behavioral analysis. Saccade latency was calculated as the time between array onset and saccade onset for no-step and noncompensated trials. For compensated trials, the saccade latency was calculated as the time between the saccade onset and T2 onset.

#### 2.2.4. Task Performance

Behavioral performance was evaluated through measurements of the proportion of noncompensated redirect trials, saccadic RT, and TSD in redirect trials. Search-step task performance can be characterized by a mathematical model of a race between independent processes that generate (GO1) and inhibit (STOP) the saccade to the initial target location [47,126]. If the GO1 process finishes first, then the saccade to T1 is executed, whereas if the STOP process finishes first, the saccade to T1 is inhibited. The latency of the GO1 process can be measured from the reaction times (RTs) of saccades to T1, but the latency of the STOP process must be estimated. The independent race model can provide an estimate of the time needed to respond to the target step and cancel the saccade to T1 (i.e., the time needed for the STOP process to complete). This duration is referred to as the *target-step reaction time* (TSRT). It is comparable to the stop-signal reaction time (SSRT) in experiment 1 [10,67,83]. The integration method was used to calculate the TSRT [47,137,138], which is the most reliable and least biased approach for calculating TSRT in paradigms that employ a dynamic tracking procedure [139]. The TSRT was computed by sorting RTs in no-step trials in ascending order for each participant. Then, from this sorted set of RTs, the RT corresponding to the proportion of noncompensated trials was identified. The mean TSD was then subtracted from this RT.

#### 2.2.5. Statistical Analyses

Two-tailed independent t-tests were used to compare the TSRT and the proportion of compensated trials between groups. A mixed-model ANOVA was used to examine the RT in the trial (no-step, compensated, noncompensated) as a within-participants factor and diagnostic group (SIB, HC) as a between-participants factor. Reported effect sizes for the ANOVAs are generalized eta squared. Greenhouse–Geisser adjustments of degrees of freedom were performed to correct for sphericity violations.

#### 2.2.6. fMRI Data Acquisition and Analysis

##### Data Acquisition

Scans were acquired on a 3.0 T Achieva MRI scanner (Philips Medical Systems) at the University Medical Center Utrecht using an eight-channel sensitivity-encoding (SENSE) parallel imaging head coil. Whole-brain T2*-weighted echo planar images with blood-oxygen-level-dependent (BOLD) contrast were acquired (4 sessions, 152 volumes, 35 slices per volume, interleaved acquisition, TR 2 s, TE 35 ms, field of view 256 × 256 × 120 mm, flip angle 70°, 96 × 96 × 35 matrix, voxel size 2.67 × 2.67 × 3.42, SENSE factor 2.4 anterior–posterior) oriented in a transverse plane were acquired. The first six images were discarded to accommodate T1 equilibration effects. A whole-brain three-dimensional fast-field echo T1-weighted scan (200 slices, TR 10 ms, TE 4.6 ms, flip angle 8°, field of view 240 × 240 ×160 mm, voxel size 0.75 × 0.8 × 0.75 mm) was acquired for within-participant registration purposes.

To account for respiratory and cardiac pulsality effects that contaminate BOLD fMRI time series, respiration and cardiac signals were measured. ECG electrodes recorded cardiac signals sampled at 500 Hz and a band wrapped around participants’ midsection recorded respiration sampled at 500 Hz.

##### Preprocessing

Functional imaging data were preprocessed and analyzed using raw fMRI data were first preprocessed spatially. Images were realigned to correct for head motion using rigid body transformations and a mean functional image was created. Next, a slice timing correction was performed by temporally interpolating the signal of each slice to the acquisition time of the middle slice. The anatomical image was co-registered to the mean functional image using the normalized mutual information criteria method. Segmentation and normalization of the anatomical image into Montreal Neurological Institute (MNI) space was achieved using the unified segmentation method [144]. The obtained normalization parameters were applied to the functional scans, which were in register with the anatomical images. Functional scans were spatially smoothed with a Gaussian kernel with a full width at half maximum (FWHM) of 6 mm. Finally, the volumes were despiked in order to remove any remaining motion-related noise using AFNI’s 3Ddespike function

##### Statistical Analyses: First-Level General Linear Models

The general linear model (GLM) framework was used for statistical analyses using a two-level procedure. First-level statistical analysis modeled the no-step, follow, compensated, and noncompensated trials at the individual level. Six 10 s rest (fixation only) trials were included in the design but were not explicitly modeled and constituted an implicit baseline. Regressors were created by convolving delta functions coding the stimulus array onset with a canonical hemodynamic response function. Twenty nuisance regressors were included to model cardiac and respiratory pulsality using the RETROICOR method with fifth-order Fourier expansions [145]. Temporal autocorrelation in the fMRI data was modeled using autoregressive modeling of the first order by prewhitening the GLM equation. Data were also high-pass filtered during prewhitening with a cutoff cycle length of 70 s.

We determined our contrasts of interest by considering how responses to the instruction to redirect would change neural processing as compared to making a visually guided saccade. Therefore, we focused our analysis on three contrasts: redirect versus fixation, no-step versus fixation, and redirect versus no-step. Combining compensated and noncompensated redirect trials into a single condition was motivated by the goal of understanding the neural basis of inhibitory control, which was presumably engaged in both the compensated and noncompensated trials. According to the race model logic, compensated trials and noncompensated trials differ in whether the initiated STOP process “won” the race. The decision to combine the compensated and noncompensated trials was justified based on prior work in our lab [99] and others [146] that reported no regions in which activity was greater in compensated trials.

##### Statistical Analyses: Second-Level General Linear Models

First-level contrasts were analyzed with a whole-brain second-level random-effects analysis using one-sample *t-*tests. Second-level contrasts were calculated for HC, SIB, and both groups combined. These contrasts are reported in an exploratory whole-brain analysis in the Supplementary Material.

**Figure 2:**
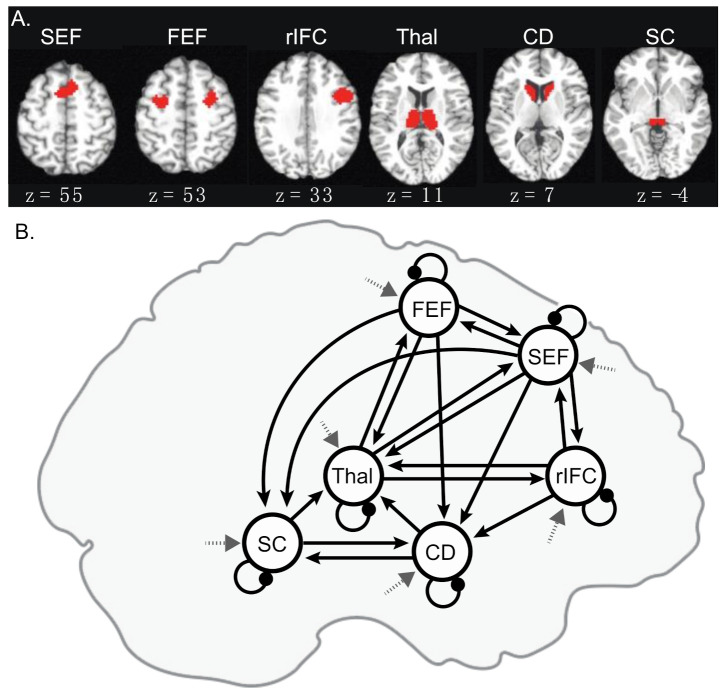
Regions of interest (ROIs) and the full model for the dynamic causal modeling analyses. (**A**) Visualization of the regions of interest with the corresponding MNI z-coordinate for each slice. (**B**) The connections that were used in the full DCM model. Between-region connections (solid arrows), inhibitory self-connections (dot tipped arrows), and driving input (grey arrows with dashed lines) were all switched “on.” The modulation of all between region connections and inhibitory self-connections was modeled. (SEF: supplementary eye field (bilateral); FEF: frontal eye fields (bilateral); rIFC: right interior frontal cortex; Thal: thalamus; CD: caudate; SC: superior colliculus).

We used a region of interest (ROI) analysis approach to identify group differences in the contrasts outlined above. Our ROIs included three cortical regions (FEF, SEF, and rIFC) and three subcortical regions (superior colliculus, caudate, and thalamus) that are all known to be involved in the inhibition of saccades in humans and non-human primates (see Figure 2A). Cortical ROI identification was guided by anatomical knowledge and defined by functional activation in the combined sample for the redirect versus no-step contrast thresholded at the uncorrected *p*-value (*p* < 1.0 × 10^−8^; a threshold at which the rIFC and insula formed two separate clusters in the right hemisphere). The bilateral FEF activation was observed and the clusters in the two hemispheres were treated as a single ROI during signal extraction. A bilateral SEF ROI was constructed in the same manner. IFC activation was characterized only in the right hemisphere, in line with previous reports [99]. To accommodate regional heterogeneity, we dilated these clusters using one voxel. Subcortical ROIs were anatomically defined and created by manually delineating these regions on averaged and normalized high-resolution T1 images from a group of 37 healthy controls, as reported in Thakkar et al. [99]. These structures were clearly visible, as the normalization procedures that are used for subcortical regions are very effective. We did not have hypotheses about functional differences in cognitive control across hemispheres in subcortical ROIs given our prior work [99], and therefore, these subcortical ROIs were combined across hemispheres. Percent signal change was extracted separately from each of the six ROIs for redirect and no-step trials. For each ROI, repeated-measures ANOVAs were conducted to investigate the effects of condition (redirect versus no-step), group (SIB versus HC), and their interaction. Significant group-by-condition interactions were followed up with independent t-tests to investigate group differences in each condition, as well as paired t-tests to investigate the effects of the condition in each group.

##### Dynamic Causal Modeling

Effective connectivity between our ROIs was assessed using the DCM framework [128,129] (DCM for fMRI using SPM12 [130]). Using this framework, we examined whether the instruction to exert executive control over planned movements modulated causal connections between and within oculomotor control regions.

DCM identifies causal influences within a network by building forward generative models of inferred neural activity [128,147,148] based on a driving input, parameterized connections between regions, self-connections, and task-related modulation. Parameters that characterize effective connections between and within regions and how the instruction to redirect a saccade modulates those connections are then iteratively optimized. For each iteration, the predicted (inferred) neural activity is compared to the time series of the experiment and then updated by adjusting parameters in the generative model. This optimization (inversion) balances the complexity (in terms of the change between each prior parameter value and the posterior estimated parameter value) and fit (between the predicted response and the observed time series) of the model. By optimizing across effective connectivity parameters, the dynamic causal influences of the neural networks can be identified.

In the present work, this computational theoretical framework is leveraged to identify effective connectivity similarities and differences between SIB and HC within a proposed oculomotor control network (including the SEF, FEF, rIFC, thalamus, caudate, and superior colliculus). Within each ROI, the voxel with the peak t-statistic from the individual-level redirect versus no-step contrasts was identified, and an 8 mm radius sphere was defined around that voxel for each participant. Then, the time series reflecting the primary eigenvector adjusted for effects of interest was extracted from the overlap of each individual’s sphere and the larger ROI used in the ROI analysis. The network connectivity (see Figure 2B) relied on known anatomical connections between regions showing saccade-related physiology in the animal literature (e.g., FEF, SEF, superior colliculus), as well as regions involved in the reactive inhibition of planned movements (e.g., rIFC and caudate) where the thalamus routes signals between these regions. Based on connections between these regions identified in the introduction, nineteen of the thirty possible between-region connections were switched “on” (solid arrows in Figure 2B), whereas other between-region connections were switched “off.” In order to properly model the excitatory/inhibitory balance within each region, self-connections reflecting self-inhibition in each region were also switched “on.” A new GLM was created to define onsets for the DCM model with a regressor for tasks that included all events in the experiment (no-step, redirect, and follow trials) that were used to provide driving input to all locations (dashed arrows in Figure 2B). A second regressor for redirect trials (both compensated and noncompensated trials) was defined and used to modulate all between-region and within-region connections given the reactive inhibition requirements of the task. The input was mean centered with inputs scaled to account for a zero duration (see [128]), which means that between-region and within-region connections (instantiated in an “A matrix”) should be interpreted as the average effective connectivity, and modulation parameters (instantiated in a “B matrix”) add or subtract from that average.

In order to optimize our model search space, a full model was inverted for each participant and then a parametric empirical Bayes (PEB) analysis (optimized over the A and B matrices together), followed by a Bayesian model comparison, was used to estimate average parameter values of each group [128,129,130]. This approach identified parameter estimates averaged across possible models and weighted by model evidence. Two results were generated for each group: the mean effective connectivity within the task (A matrix) and the modulation due to the instruction to redirect the planned saccade (B matrix). This allowed for the characterization of effective connectivity underlying executive control over saccade inhibition for each group.

Next, to assess the commonalities and differences in the groups’ effective connectivity, we used a second-level PEB with all individual inverted DCMs (HC participants were coded as −1 and SIB were coded as 1). Again, average parameter estimates were identified using a Bayesian model comparison. This approach yielded four results: overall mean effective connectivity across both groups (A matrix), mean modulation across both groups (B matrix), group differences in mean effective connectivity (differences in A matrices), and group differences in mean modulation due to the instruction to redirect (differences in B matrices).

We interpreted the DCM modeling results by focusing on the parameters with posterior probabilities greater than 95% (labeled here as “credible”). Credible positive values (“excitatory” connections) for between-region mean effective connectivity parameters indicate that increased activity in a source region leads to an increased change in the activation in the receiving region, whereas credible negative parameter values (“inhibitory” connections) indicate effective connectivity such that increased activity in the source region cause decreased changes in the activation in the receiving region. Self-connections reflect within-region inhibition with initial (default) parameter values of −0.5 and positive reported parameters indicate more inhibition, whereas negative parameters reflect less self-inhibition than this starting value. Modulation parameters reflect additive changes in effective connectivity on redirect trials relative to the mean effective connectivity.

Finally, we examined whether the oculomotor control network effective connectivity and the TSRT were related. Connections with credible group differences (both mean effective connectivity and modulation on redirect trials) were identified in the second-level PEB analysis. Then, these parameters were extracted from individual optimized DCM models that were returned from the group PEB analyses. Extracted parameters were put into a backward elimination linear regression in SPSS, separately for each group. A backward elimination procedure removed non-significant parameters until a final model was found for each group that best predicted the TSRT.

## 3. Results

### 3.1. Experiment 1

#### 3.1.1. Behavioral Data

The behavioral data are summarized in Table 3.

##### Probability of Stop-Signal Trial Inhibition

The dynamic tracking procedure to adjust the SSD succeeded given that participants failed to cancel their saccade on roughly half the stop-signal trials. The mean percentage of noncanceled stop-signal trials was 48.86% and there was no group difference (t(24) = 1.05, *p* = 0.305).

##### Speed of Response Execution

**Figure 3:**
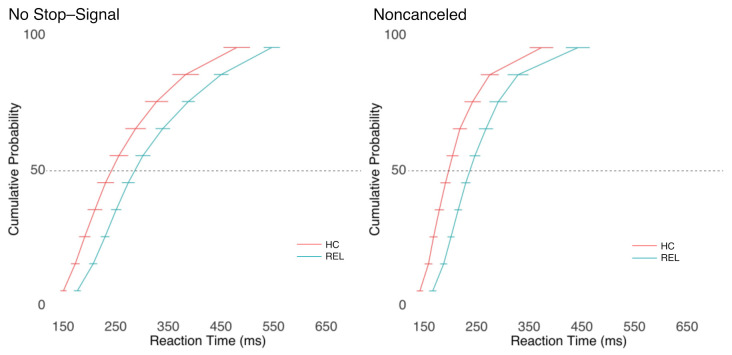
Vincentized RT distributions for no stop-signal trials (**left panel**) and noncanceled stop-signal trials (**right panel**) for experiment 1. The reaction times for each participant were binned into deciles split by trial type in order to visualize the distributions, which complimented the ANOVAs examining the mean reaction times that are reported in the main text. The decile means were averaged across participants for each group (REL and HC) to generate the distributions. Each error bar around each decile is the standard error of the mean RT within that decile.

Figure 3 shows the cumulative distributions of RTs. The ANOVAs comparing the mean RT per person in each condition revealed a significant effect of trial type in the RT (F(1, 24) = 117.92, *p* < 0.001, η^2^ = 0.34). Consistent with the race model logic, noncanceled trials (i.e., those that escaped inhibition) were faster than no stop-signal trials. There was also a significant main effect of group (F(1,24) = 8.63, *p* = 0.007, η^2^ = 0.24), with REL responding slower than HC. There was no significant group-by-trial-type interaction (F(1,24) = 0.25, *p* = 0.620, η^2^ < 0.01).

##### SSRT

The SSRT was significantly longer in REL than HC (t(24) = 2.40, *p* = 0.024; see Table 3).

### 3.2. Experiment 2

#### 3.2.1. Behavioral Data

See Table 4 for a summary of the behavioral data.

##### Percent Compensated

The dynamic tracking procedure succeeded, as participants did not compensate for the target location change in roughly half the redirect trials. The mean percentage of noncompensated trials was 45.56% and there was no group difference (t(43) = 0.99, *p* = 0.327).

##### Speed of Response Execution

**Figure 4:**
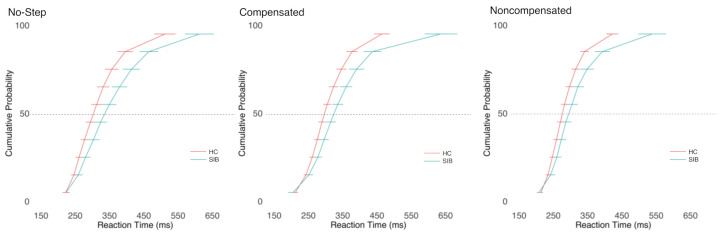
Vincentized RT distributions for no-step trials (**left panel**), compensated trials (relative to T2 onset; (**middle panel**)), and noncompensated trials (**right panel**) for experiment 2. The reaction times for each participant were binned into deciles split by trial type in order to visualize the distributions, which complimented the ANOVAs examining the mean reaction times reported in the main text. Decile means were averaged across participants for each group (HC and SIB). Each error bar shows the standard error of each decile mean.

Figure 4 depicts the cumulative RT distributions. ANOVAs comparing the mean RT per person in each condition revealed a significant effect of trial type on the RT (F(1.98, 84.99) = 15.04, *p* < 0.001, η^2^ = 0.05). Follow-up paired t-tests showed that, consistent with the race model logic, noncompensated trials were significantly faster than both no-step (t(44) = 5.49, *p* < 0.001) and compensated (t(44) = 3.63, *p* < 0.001) trials. There was no difference between the RTs in the no-step and compensated trials (t(44) = 1.57, *p* = 0.938). SIB tended to respond more slowly than HC, but the group difference did not reach statistical significance (F(1,43) = 3.02, *p* = 0.089, η^2^ = 0.06). There was no significant group-by-trial-type interaction (F(1.98,84.99) = 0.31, *p* = 0.731, η^2^ < 0.01).

##### TSRT

The TSRT tended to be longer in SIB relative to HC, although the group difference did not reach statistical significance (t(43) = 1.94, *p* = 0.058; see Table 4).

#### 3.2.2. fMRI Data

##### General Linear Modelling

**Figure 5:**
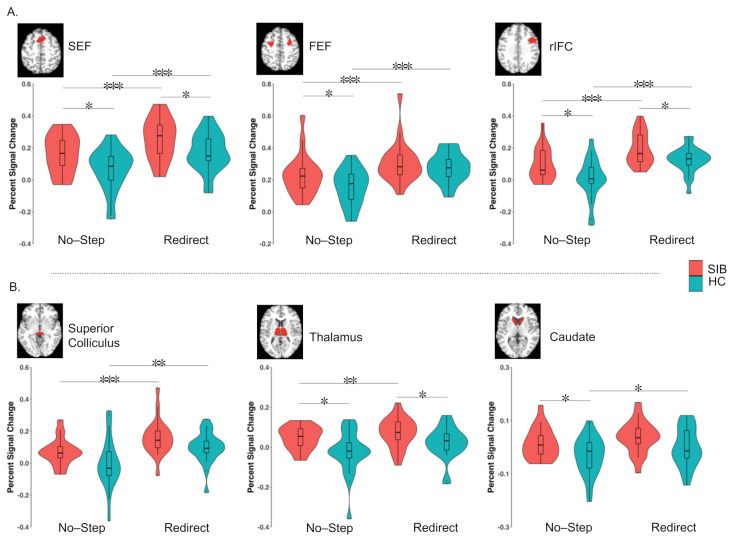
Violin plots of percent signal change for the redirect and no-step trials in (**A**) cortical and (**B**) sub-cortical regions of interest for the SIB and HC groups. Boxplots depict the median and quartile values. Outliers with values beyond two interquartile intervals from the median appear as individual dots. Visualized significant differences reflect paired *t*-tests for within group comparisons and independent t-tests for between-group comparisons (* *p* < 0.05, ** *p* < 0.01, *** *p* < 0.001). SEF: supplementary eye fields (bilateral); FEF: frontal eye fields (bilateral); rIFC: right interior frontal cortex.

Figure 5 depicts the results from the ROI analyses. We identified the cortical regions based on the significant whole-brain-level main effects of condition. Thus, all three cortical regions (bilateral SEF, bilateral FEF, and rIFC) showed the main effects of condition (all p’s < 0.001). Paired t-tests showed significantly more activation in the redirect trials than the no-step trials across both groups in all three cortical regions (all p’s ≤ 0.001). There was a significant main effect of the group in the SEF (F(1,41) = 6.09, *p* = 0.018, η^2^ = 0.12) and the rIFC (F(1,41) = 7.22, *p* = 0.010, η^2^ = 0.13), with SIB showing greater activation than HC. Although there was no main effect of group in the FEF (F(1,41) = 2.46, *p* = 0.125, η^2^ = 0.05), there was a significant group-by-condition interaction (F(1,41) = 4.94, *p* = 0.032, η^2^ = 0.01). SIB had greater FEF activation than HC for no-step trials (t(38) = 2.04, *p* = 0.049), but not redirect trials (t(38) = 1.37, *p* = 0.18); thus, there was reduced differential activation between redirect and no-step trials in SIB. Significant group-by-condition interactions were not observed in either the SEF or rIFC.

In our subcortical ROIs, we found a significant main effect of condition in the superior colliculus (F(1,41) = 39.77, *p* < 0.001, η^2^ = 0.14), caudate (F(1,41) = 11.76, *p* < 0.001, η^2^ = 0.05), and the thalamus (F(1,41) = 13.15, *p* = 0.001, η^2^ = 0.05), with greater activation in redirect trials as compared to no-step trials. There were significant main effects of group in the caudate (F(1,41) = 5.43, *p* = 0.025, η^2^ = 0.10), thalamus (F(1,41) = 7.11, *p* = 0.011, η^2^ = 0.13), and the superior colliculus (F(1,41) = 4.15, *p* = 0.048, η^2^ = 0.08). SIB showed more activation than HC in all regions. There were no significant group-by-condition interactions in any of the subcortical ROIs (all p’s > 0.272).

Results from the exploratory whole-brain analyses are described in the Supplementary Materials (visualized in Figure S1, with significant results reported in Table S1).

##### Dynamic Causal Modeling

**Figure 6:**
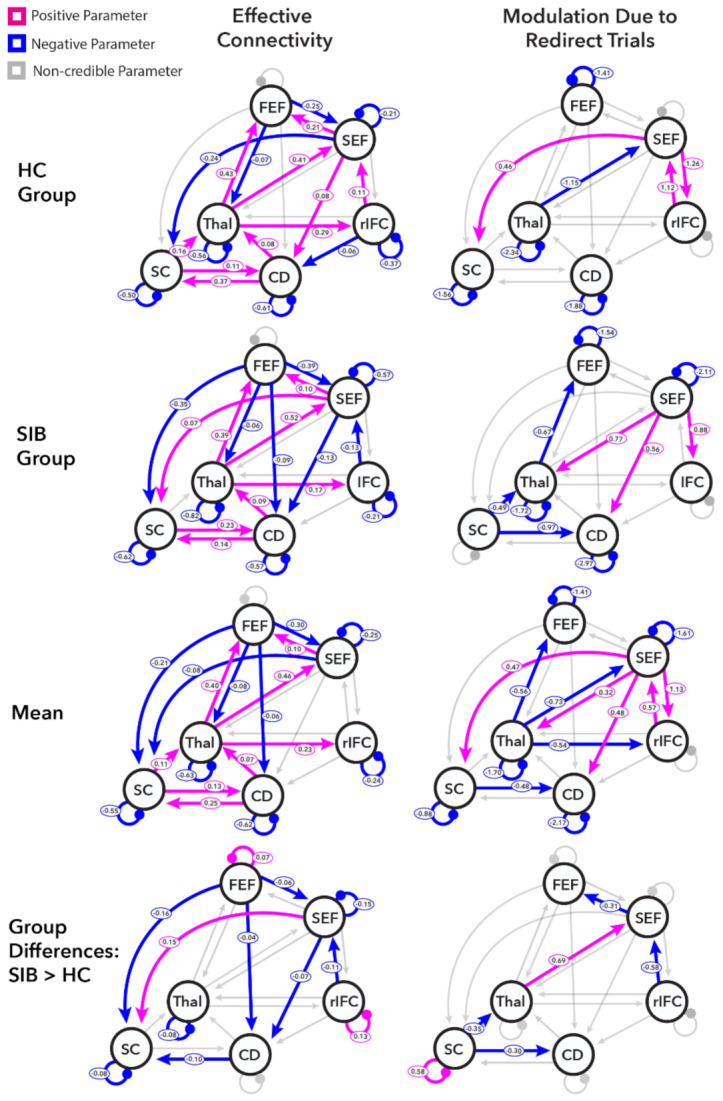
Results of the dynamic causal modeling (DCM) representing separate parametric empirical Bayesian analyses and the Bayesian model comparison for HC and SIB, which are depicted in the top two rows, respectively. The bottom two rows depict the group data. The left column (effective connectivity) depicts the mean effects throughout the task, whereas the right column (modulation due to redirect trials) refers to parameters that additively modulate the mean connectivity on redirect trials. The mean row depicts the group average effective connectivity and modulation in the redirect trials across both groups. The group differences row depicts the connections with credible between-group differences. The credible parameters were those with a posterior probability of differing from zero greater than 95%. SEF: supplementary eye fields (bilateral); FEF: frontal eye fields (bilateral); rIFC: right interior frontal cortex; Thal: thalamus; CD: caudate; SC: superior colliculus).

Figure 6 shows the results of the DCM analyses for the HC (top row) and SIB (second row) groups, as well as the group analysis including the mean across groups (third row) and group differences (bottom row). Each analysis was broken down into the mean effective connectivity (left column) and the modulation by the instruction to redirect (right column). Supplementary Tables S2–S4 present all parameter estimates and their associated posterior probabilities.

The group commonalities that were identified in the second-level PEB (third row, first column of Figure 6) suggest the network of regions selected was reliably engaged in both groups throughout the task. Self-inhibition was reduced below the initial default values in all regions except the FEF. The FEF had widespread inhibitory effects on regions, including the SEF, caudate, thalamus, and superior colliculus. The SEF excited the FEF and inhibited the superior colliculus. The caudate showed excitatory connections to the thalamus and the superior colliculus, and the thalamus showed widespread excitation of the FEF, SEF, and rIFC. The superior colliculus had excitatory effects on both the thalamus and caudate. These connections were modulated by the instruction to redirect the participant’s gaze. The within-region parameters were modulated in all regions, except the rIFC, such that the instruction to redirect resulted in reductions in self-inhibition across groups. Additionally, on redirect trials, the influence of the SEF on the superior colliculus, caudate, thalamus, and rIFC across groups was positively modulated. On the other hand, the instruction to redirect had an inhibitory influence on the connections from the thalamus to the FEF, SEF, and rIFC, as well as from the superior colliculus to the caudate.

There were group differences in both the effective connectivity and modulation parameters identified in the second-level PEB (fourth row, Figure 6). The mean connectivity differences were seen in both the inhibitory self-connections and the between-region connections. HC showed less self-inhibition of the FEF and rIFC than SIB, whereas the SEF, thalamus, and superior colliculus showed more self-inhibition for HC than SIB. The between-region mean connectivity showed group differences in connections from the FEF: although FEF had an inhibitory influence on the SEF, superior colliculus, and caudate in both groups, this inhibitory influence was more prominent in SIB. The caudate showed an excitatory influence on the superior colliculus in both groups but to a lesser extent in SIB. The rIFC showed an excitatory influence in the connection to SEF among HC, but this connection was inhibitory among SIB. The same pattern of group differences was observed in the connection from SEF to the caudate. HC showed inhibition from SEF to the superior colliculus, but in SIB, this connection was excitatory.

Group differences were also observed in the degree to which effective connectivity was modulated by the instruction to inhibit. In the superior colliculus, HC showed reduced modulatory self-inhibition, whereas SIB showed the baseline amount of self-inhibition. This difference led to credibly more modulatory self-inhibition in the superior colliculus in redirect trials among SIB. Further, redirecting a saccade did not inhibit the connections from the superior colliculus to the thalamus and the caudate among HC, whereas these connections were inhibited among SIB. The connection from the thalamus to the SEF was negatively modulated by the instruction to inhibit in HC but did not differ from zero for SIB. Conversely, the connection from the rIFC to the SEF had excitatory modulation for HC but did not differ from zero for SIB. Neither group showed credible modulation of the connection from the SEF to the FEF separately; however, the second-level PEB credibly identified more inhibition among siblings. This was due to HC exhibiting no modulation of this parameter at the group level, whereas SIB showed a non-credible inhibitory modulation on redirect trials. Note that the Bayesian model averaging step included a model comparison procedure where parameters were “turned off” by setting them to zero and left off if the model was fit better without them. The BMA that was returned in the HC analysis suggested a value of 0 with a probability of 0 for the modulation from the connection between the SEF to FEF, indicating that the model was a better fit without this parameter.

Our final aim was to evaluate whether and how the strength of effective connectivity parameters within the oculomotor control network were related to the TSRT. The mean and modulation parameters showing credible group differences were used to predict the TSRT using stepwise backward elimination within each group. A model for HC was identified that significantly predicted the TSRT (F(9,13) = 4.26, *p* = 0.009, adjusted R^2^ = 0.57; see Table 5) based on the combination of eight mean effective connectivity parameters and one modulatory parameter. More positive parameters in the connections from the FEF to the SEF, the SEF to the caudate, and the rIFC to the SEF predicted shorter TSRTs. Conversely, more positive parameters from the SEF to the superior colliculus and from the caudate to the superior colliculus were related to longer TSRTs. More self-inhibition in the rIFC was associated with reduced TSRTs, whereas more self-inhibition in the FEF and more self-inhibition in the thalamus were associated with longer TSRTs. More positive modulation of the connection from the thalamus to the SEF was also associated with longer TSRTs. A model for SIB was identified that significantly predicted the TSRT (F(1,18) = 4.80, *p* = 0.042, adjusted R^2^ = 0.17; see Table 5) based on the mean effective connectivity between the FEF and the caudate. More positive effective connectivity parameters were associated with longer TSRTs. Given that these parameters were selected based on exhibiting credible group differences and that we observed marginal group differences in the TSRT behaviorally, we report the results of exploratory regressions predicting TSRTs based on group, parameter values, and group-by-parameter interactions in the Supplementary Materials. These regression analyses were done separately for each parameter value that predicted TSRTs in either HC or SIB.

## 4. Discussion

Inhibitory control over planned actions in the context of updated goals and changing environmental demands is a critical cognitive function that involves both proactive and reactive processes. Less efficient inhibitory control processes were described in individuals with schizophrenia [18]. In the current study, we sought to examine whether inefficient cognitive control of movement may reflect familial vulnerability to schizophrenia. We measured performance on a task requiring rapid stopping or changing of a saccade plan in unaffected first-degree relatives of individuals with schizophrenia and healthy controls and examined group differences in the functional architecture supporting rapid inhibition and the modification of saccade plans. Across two experiments, we found data that were suggestive of less efficient inhibitory control (i.e., longer SSRT/TSRT) and slower RTs for visually guided saccades (i.e., on GO trials) in REL. In our second experiment, we identified functional activity and effective connectivity within a network of brain regions that are associated with oculomotor control. In most of our regions of interest, siblings of individuals with schizophrenia showed greater activation than healthy controls, both in trials that required a saccade plan to be inhibited and modified (redirect trials) and in trials that simply required a visually guided saccade. Finally, we identified group differences in mean effective connectivity across the task and modulation of effective connectivity resulting from movement inhibition demands of redirect trials within the oculomotor control network. These results and their implications for understanding the nature of inhibitory control impairments in schizophrenia are discussed in turn.

The behavioral results across the two studies are suggestive of group differences between unaffected relatives and healthy controls and provide converging initial evidence regarding the size and direction of performance differences between healthy controls and unaffected relatives in variants of the saccadic stop-signal task. Experiment 1 showed significantly longer RTs and SSRTs among unaffected relatives. Similar results were observed in experiment 2, although these results only approached significance (see the Supplementary Materials for a Bayes factor analysis of these results that suggests there was weak or anecdotal evidence supporting longer SSRTs/TSRTs in unaffected relatives). The less robust group differences and overall slower oculomotor RT in experiment 2 may have been due to task differences: in the stop-signal task (experiment 1), participants must inhibit an action outright, whereas in the search-step task (experiment 2), participants must inhibit an initial response and plan a saccade to a new target. Differences may also be attributable to sample differences. Of note, however, is that neither age, IQ, nor social functioning differed significantly between the two unaffected relatives groups (see the Supplementary Materials). We do report IQ differences between relatives and healthy controls that approached significance (see Table 1) in experiment 1, whereas IQ was more closely matched in experiment 2. However, prior studies have not found relationships between the SSRT and IQ [4,19,57], which suggests that small group differences in IQ are unlikely to explain our current findings.

Bayesian modeling would suggest that longer SSRTs and marginally longer TSRTs among relatives indicate a slower inhibitory process, a failure in initiating an inhibitory process in response to the cue, or both [149]. Although longer SSRTs/TSRTs in unaffected relatives mirror our findings in individuals with schizophrenia [67,68,69], they are inconsistent with previous studies that have not reported significantly longer SSRTs in unaffected relatives using a manual version of the stop-signal task [5,9,60,66,70,71,74]. Differences between these studies and the current results are potentially explained by response modality [150]. Unlike hand movements, saccades are ballistic and, thus, are less readily modifiable during the execution stage. Hand and eye movements also have different ecological relevance, as hand movements allow for interaction with and manipulation of the external world. Finally, there is evidence for separable central and peripheral inhibitory mechanisms [75], which differ in the extent to which they are effective at stopping eye versus manual movements. Thus, the saccade version of the stop-signal tasks and their variants may provide a more sensitive measure of inhibition that is relevant for the schizophrenia phenotype. In contrast to our findings in individuals with schizophrenia who did not show RT differences relative to healthy controls [69], unaffected relatives took more time to initiate a response. While this may indicate impairments in saccade generation, this interpretation is not supported by previous literature that reports unimpaired reflexive saccade kinematics in unaffected relatives [151]. Instead, slower RTs may reflect compensatory strategic slowing among unaffected relatives, that is, adopting a waiting strategy. This interpretation is potentially bolstered by our neuroimaging results.

The behavioral differences between unaffected siblings and healthy controls in experiment 2 were accompanied by the brain activation differences between groups. Consistent with our prior work [69,99], both healthy controls and unaffected siblings showed greater activation in redirect trials as compared to no-step trials in all regions of our oculomotor control network. Unaffected siblings showed more activation than healthy controls across conditions in most ROIs within our network: the SEF, rIFC, caudate, superior colliculus, and thalamus across conditions. In addition, a group-by-condition interaction was observed in the FEF: the two groups activated the FEF to a similar extent in redirect trials, but siblings had increased activation on no-step trials. Generally speaking, siblings of individuals with schizophrenia showed broad increases in activation in a network of regions supporting inhibition over a planned eye movement. Like with the behavioral data, these data from unaffected siblings show overlaps and distinctions from our findings in individuals with schizophrenia performing the same task during fMRI [69]. Individuals with schizophrenia generally showed a group-by-condition interaction in this network such that they engaged these regions more than healthy controls in no-step trials but to an equal magnitude as controls in redirect trials, resulting in smaller differences in activation in redirect versus no-step trials. Thus, although both individuals with schizophrenia and unaffected siblings activated this network more in no-step trials, siblings also activated this network more on redirect trials where individuals with schizophrenia did not. Importantly, the siblings in this study were unmedicated, which rules out potential confounds due to psychotropic medication on oculomotor control. One may speculate that increased activation mirrors the RT data, where relatives are adopting compensatory proactive processes that result in longer RTs and greater activation related to inhibitory processes. We acknowledge, however, that interpreting these broad increases in activity in siblings is complicated by the heterogeneity of neuronal populations within these regions (e.g., fixation and movement neurons in the FEF) and the variety of processes involved in redirecting an eye movement that are not related to inhibition (e.g., target selection, movement preparation to competing saccade targets).

DCM analyses provide further insights into the neural underpinnings of gaze control in unaffected relatives and healthy controls. Before interpreting these effective connectivity results, we first review how nodes in this network interact to exert control over planned gaze shifts. The FEF and superior colliculus contain populations of fixation and movement cells [80,81,82,83,84]. In non-human primates performing the stop-signal task, activity in movement cells attenuates their firing upon presentation of the stop-signal. If that attenuation is sufficiently fast and robust, the saccade is successfully canceled; thus, modulation of movement cells in the FEF and superior colliculus must occur in order for the planned saccade to be successfully canceled. A decrease in saccade-related activity in the FEF and superior colliculus can be instantiated in several ways. First, movement cells may be inhibited by fixation cells; indeed, while movement cell activity decreases upon stop-signal presentation, fixation cell activity ramps up [79,80]. Alternatively, movement activity may be modulated by neurons outside of the FEF and superior colliculus. For example, signals from the basal ganglia can inhibit movements via the indirect pathway that projects from the striatum to the substantia nigra pars reticulata, which then increases its inhibition of the superior colliculus directly and of cortical movement areas via the thalamus [84]. These basal ganglia nuclei (including the striatum) receive projections from the supplementary motor complex and IFC, and human neuroimaging, electrocorticographical, and neurostimulation work suggested that these cortical inputs to the basal ganglia are central to effective performance in the stop-signal task [103,152,153,154,155]. Primate neurophysiology suggests that modulation of activity in the SEF following a stop-signal is too late for it to play a direct role in the control of movement [87,88,89]. Instead, the SEF may exert proactive control over movement cells in the FEF and superior colliculus directly [87,90,91] or via the basal ganglia [92,93] based on recent performance, co-activation of competing response plans, or computations of error costs [89,156,157]. In sum, there are several ways in which movement activity in the FEF and superior colliculus may be modulated, involving interactions within these regions or signals from external regions, such as the SEF, caudate, and rIFC.

The DCM analyses showed that across groups, the selected oculomotor control network was reliably engaged during the task, which is consistent with a previous study in individuals with schizophrenia [69]. Furthermore, in HC, several of the mean effective connectivity parameters were related to TSRT, indicating their relevance for efficient control. The DCM analysis also revealed group differences, both in the mean effective connectivity and the degree to which the instruction to inhibit and redirect a planned gaze shift modulated the effective connectivity of these connections. The task-wide activation differences between groups seen in the GLM analysis are at least partly recapitulated in our DCM analysis, which suggests that the task differentially engages key inhibitory mechanisms among siblings. We saw group differences in the mean effective connectivity such that siblings show more inhibitory effective connectivity than healthy controls from the FEF to the SEF, to the superior colliculus, and to the caudate. This widespread increased inhibition might reflect less propagated information about activity in the visual, movement, and fixation neurons in the FEF, which is information that the SEF uses to assess trial-by-trial proactive control demands [158] and that facilitates movement plan formation in the superior colliculus [159] and the caudate [84]. The mean effective connectivity from the FEF to the caudate was the only parameter that was significantly associated with TSRTs in siblings. Siblings showed more inhibition from the FEF to the caudate, and greater inhibition was related to faster TSRTs, suggesting that siblings may be relying more on this pathway across the task to support efficient inhibition of movement plans than HC. We also saw less excitation from the caudate to the superior colliculus in unaffected siblings, perhaps reflecting group differences in the recruitment of the indirect pathway throughout the task [84,85,86]. Another indication that inhibitory control might have been engaged differentially throughout the task among unaffected siblings was the different connectivity patterns from the rIFC to the SEF and from the SEF to the caudate, which were inhibitory in siblings and excitatory in controls. These connections were previously highlighted as a key inhibitory pathway [93,100,103,104,152,153,160] and, interestingly, individuals with schizophrenia showed a similar pattern of differential effective connectivity relative to healthy controls in our prior work [69]. Siblings also showed excitation from the SEF to the superior colliculus, whereas controls showed inhibition. This may reflect an inappropriate engagement of proactive inhibitory control processes in the SEF across trials. Finally, we found group differences in self-inhibition such that siblings showed more self-inhibition in the FEF and the rIFC, but less self-inhibition in the SEF, thalamus, and superior colliculus. Altered self-inhibition of these regions was also observed in individuals with schizophrenia in a previous study [69]. Three of these self-inhibition parameters (thalamus, superior colliculus, and rIFC) were associated with TSRTs across participants, suggesting that these altered within-region dynamics related to task performance. These group differences in self-inhibition parameters may indicate altered excitatory/inhibitory balance in these regions, which is in line with prior findings in relatives of patients with psychosis [161,162]. Altered inhibitory interneuron function and the resulting excitatory/inhibitory imbalance were proposed as a pathophysiological mechanism in schizophrenia [163,164]. The group comparisons of activation patterns and mean task-related effective connectivity, therefore, suggested that siblings showed systematic differences in how regions that are generally involved in task performance are activated and connected within the oculomotor network.

Group differences in how the instruction to redirect a planned eye movement modulated these connections were also identified. In the first-level PEBs, siblings showed non-credible inhibitory modulation of the connection from the SEF to the FEF, whereas controls showed no modulation of this parameter. This difference was credible in the second-level PEB. This may reflect differences in expectations that the SEF propagates to the FEF that adjust the predicted trial-by-trial inhibitory demands [88]. Siblings also showed less excitatory modulation of the connection between the rIFC and the SEF, perhaps reflecting reduced inhibitory signaling [94,165,166] or reduced influence of the detection of contextual relevance of stimuli on motor planning [167,168]. In addition, we found more inhibitory modulation among siblings in connections from the superior colliculus to the caudate and the thalamus. The modulation of the connection from the superior colliculus to the thalamus may suggest reduced afferent signals from the superior colliculus to the cortex that convey updated saccade plans; this is an idea that aligns with siblings failing to show inhibitory modulation in the connection from the thalamus to the SEF in redirect trials, as feedback from superior colliculus is routed through the thalamus [115]. This connection from the superior colliculus to the thalamus also represents a shared finding of modulatory effective connectivity in individuals with schizophrenia who show similar alterations compared to healthy controls [69]. Alterations in the connection from the superior colliculus to the caudate may reflect the abnormal engagement of saccade target selection or action inhibition mechanisms, as both types of information are conveyed through this route [120]. Finally, we saw no reduction in self-inhibition within the superior colliculus among siblings in redirect trials, which may reflect a failure to properly disinhibit a replanned saccade to the second target location. These results broadly indicate differences in how connections between regions that implement gaze control, particularly those involving the superior colliculus and the SEF, are differentially modulated in trials in which inhibitory control is required among unaffected siblings.

In sum, both behavior and functional neuroanatomy suggest differences between relatives and controls, including longer SSRTs/TSRTs in the context of overall saccade slowing, increased activity in the saccade control network, and differences in the effective connectivity throughout the task in pathways that are associated with inhibitory control. The findings of overall saccade slowing during this task, as well as what might be interpreted as over-engagement of brain regions playing a crucial role in response inhibition throughout the task, may suggest that unaffected relatives showed a proactive strategy during the task—that is, engaging inhibitory processes when they are not required by the immediate task demands (i.e., when there is no instruction to cancel a movement). Such over-engagement of inhibitory processes may reflect a compensatory strategy in the context of reactive control impairment or an overestimation of inhibitory demands (i.e., overestimating stop/step signal probability). At first glance, such an interpretation appears to be at direct odds with the previous findings showing that unaffected relatives show a blunted parametric increase in neural activity with increases in stop-signal probability, which is consistent with the reduced engagement of proactive control mechanisms [9,74,125]. However, a parsimonious explanation is that the engagement of inhibitory processes in unaffected relatives is less influenced by the overall task structure. Muddying a direct comparison between these previous studies and the current study are response modality (eyes versus hands) and working memory demands. In the aforementioned previous studies, the stop-signal probability was indicated by a cue that was presented at the beginning of the trial. That is, the probability that the trial would require control over a planned movement was explicit, and the task required maintaining the cue-probability mapping in the participant’s working memory. In the current study, the estimation of the stop-signal probability in each trial was implicit and the working memory demands were minimal. Parametric manipulation of the probability of having to cancel a saccade in future studies using saccadic stop-signal tasks may further illuminate whether the effects seen in the current study reflect alterations in the proactive engagement of inhibitory control among unaffected relatives of individuals with schizophrenia.

There are several limitations in the current work. First, the sample sizes were small and we were not able to verify the proband diagnosis for all participants in experiment 1. Thus, the current results should be viewed as exploratory. Importantly, however, we did observe similar patterns of task performance across two separate samples across the two experiments. Second, our selected oculomotor control network excluded several brain regions that are likely to be involved, either directly or indirectly, in oculomotor control. Additional basal ganglia subregions, the dorsolateral prefrontal cortex, and the superior parietal lobe are all likely to be involved in the task. A more complete network that included such regions was not selected to reduce the model space and due to limited reliable signals in small, deep brain structures.

## 5. Conclusions

Combined, these results suggest that impairments in the rapid control over a planned eye movement may reflect familial vulnerability for schizophrenia. Although future studies with larger sample sizes are necessary to draw firm conclusions, longer TSRTs/SSRTs for eye movements may serve as a potential endophenotypic marker of schizophrenia diathesis. While unaffected relatives and individuals with schizophrenia both show evidence for longer TSRTs/SSRTs on oculomotor stop-signal task and their variants, relatives show evidence for a compensatory proactive waiting strategy that is accompanied by more robust responses in task-relevant regions across conditions and a different pattern of effective connectivity across the task that is modulated differently by the instruction to exert control over a planned saccade. Given the relationship between the TSRT and these connectivity parameters, we may surmise that individuals at familial risk for schizophrenia are engaging an oculomotor control network differently than controls and in a way that compromises inhibition efficiency.

## Figures and Tables

**Table 1:** Demographic information.

	HC (*n* = 14) Mean (s.d.)	REL (*n* = 12) Mean (s.d.)	Statistic	*p*-Value
Age	36.5 (11.01)	36.5 (8.64)	t < 0.01	>0.999
Sex	7 F/7 M	7 F/5 M	χ^2^ = 0.00	0.976
IQ ^1^	108.20 (6.04)	102.48 (11.52)	t = 2.01	0.056
Handedness ^2^	0.62 (0.69)	0.73 (0.32)	t = 0.51	0.614
Education ^3^	15.79 (1.76)	15.17 (3.69)	t = 0.56	0.581

^1^ The Adult North American Reading Test (ANART [132,133]) or Wechsler Abbreviated Scale of Intelligence (WASI) was used to assess IQ. ^2^ Based on the Edinburgh Handedness Inventory; the score ranged from 0 indicating total left-handedness to 1 indicating total right-handedness. ^3^ Years of education.

**Table 2:** Demographic information.

	HC (*n* = 23) Mean (s.d.)	SIB (*n* = 22) Mean (s.d.)	Statistic	*p*-Value
Age	31.91 (8.23)	31.09 (5.46)	t = 0.40	0.694
Sex	10 F/13 M	7 F/15 M	χ^2^ = 0.25	0.618
IQ ^1^	99.72 (14.36)	100.27 (14.67)	t = 0.63	0.532
Handedness ^2^	0.79 (0.53)	0.87 (0.30)	t = 0.12	0.906
Education ^3^	6.87 (1.63)	6.32 (1.67)	t = 1.12	0.270

^1^ Based on the Nederlandse Leestest voor Volwassenen. ^2^ Based on the Edinburgh Handedness Inventory; a score of 0 indicated complete left-handedness whereas 1 indicated complete right-handedness. ^3^ Education categories: 0 = <6 years of primary education, 1 = finished 6 years of primary education, 2 = 6 years of primary education and low-level secondary education, 3 = 4 years of low-level secondary education, 4 = 4 years of average-level secondary education, 5 = 5 years of average-level secondary education, 6 = 4 years of secondary vocational training, 7 = 4 years of high-level professional education, 8 = university degree.

**Table 3:** Stop-signal task performance for healthy controls and relatives.

	HC Mean (s.d.)	REL Mean (s.d.)	Statistic	*p*-Value
No Stop-Signal Reaction Time (ms)	269.40 (50.03)	316.83 (34.91)	t(24) = 2.76	0.011
Noncanceled Reaction Time (ms)	215.72 (36.89)	257.94 (39.23)	t(24) = 2.83	0.009
SSRT (ms)	99.01 (35.38)	135.67 (42.67)	t(24) = 2.40	0.024
Probability of Inhibition (%)	49.62 (3.68)	47.97 (4.34)	t(24) = 1.05	0.305

SSRT: stop-signal reaction time.

**Table 4:** Search-step task performance.

	HC Mean (s.d.)	SIB Mean (s.d.)	Statistic	*p*-Value
No-Step Reaction Time (ms)	324.42 (78.26)	360.61 (90.95)	t(43) = 1.43	0.159
Compensated Reaction Time (ms)	312.05 (57.69)	351.07 (78.20)	t(43) = 1.91	0.063
Noncompensated Reaction Time (ms)	289.71 (48.41)	317.67 (72.27)	t(43) = 1.53	0.133
TSRT (ms)	153.59 (19.53)	169.57 (33.99)	t(43) = 1.94	0.058
Percent Noncompensated (%)	46.48 (6.09)	44.59 (6.64)	t(43) = 0.99	0.327

TSRT: target-step reaction time.

**Table 5:** Final regression model from the backward elimination procedure predicting TSRTs within each group using standardized DCM parameters showing credible group differences.

Controls: Fixed Effects	Estimate	Std. Error	t-Stat	*p*-Value
Intercept	612.38	119.82	5.11	<0.001
Mean FEF to SEF	−219.33	78.17	−2.81	0.015
Mean SEF to caudate	−3089.15	875.85	−3.53	0.004
Mean rIFC to SEF	−816.58	224.40	−3.64	0.003
Mean SEF to superior colliculus	462.38	129.99	3.56	0.004
Mean caudate to superior colliculus	911.54	281.31	3.24	0.006
Mean self-inhibition of rIFC	−1301.16	426.18	−3.05	0.009
Mean self-inhibition of FEF	712.08	258.45	2.76	0.016
Mean self-inhibition of thalamus	1367.23	321.19	4.26	<0.001
Modulation of thalamus to SEF	44.87	12.23	3.67	0.003
**Siblings: Fixed Effects**	**Estimate**	**Std. Error**	**t-Stat**	***p*-Value**
Intercept	206.01	18.16	11.34	<0.001
Mean FEF to CD	420.18	191.85	2.19	0.042

## Supplementary Materials

The following are available online at https://www.mdpi.com/article/10.3390/brainsci11091228/s1, Figure S1: Exploratory Whole Brain fMRI Analysis, Table S1. GLM results, Table S2. Control DCM parameters, Table S3. Sibling DCM parameters, Table S4. Group DCM analysis, Table S5. Regressions examining the relationship between DCM parameters and TSRT within both groups.
