# Supplementary material for: Altered Effective Connectivity within an Oculomotor Control Network in Unaffected Relatives of Individuals with Schizophrenia
Source: Brain Sci. 2021 Sep 17;11(9):1228. doi: 10.3390/brainsci11091228 (PMC8467791; doi:10.3390/brainsci11091228)
Supplement: Supplementary file 1 [file brainsci-11-01228-s001.zip › brainsci-1338856-supplementary.pdf]

Supplementary Materials

Article

Altered Effective Connectivity within an Oculomotor Control Network in Unaffected Relatives of Individuals with Schizophrenia

Matthew Lehet [1], Ivy F. Tso [2], Sohee Park [3], Sebastiaan F. W. Neggers [4], Ilse A. Thompson [4], Rene S. Kahn [5] and Katharine N. Thakkar [1,6,*]

[1] Department of Psychology, Michigan State University, East Lansing, MI 48824; USA; lehetmat@msu.edu
[2] Department of Psychiatry, University of Michigan, Ann Arbor, MI 48109, USA; ivytso@med.umich.edu
[3] Department of Psychology, Vanderbilt University, Nashville, TN 37235, USA; sohee.park@vanderbilt.edu
[4] Department of Psychiatry, University Medical Center Utrecht; Utrecht, 3584 CX; Netherlands; B.Neggers@umcutrecht.nl (S.F.W.N.); ilsethompson@hotmail.com (I.A.T.)
[5] Department of Psychiatry, Icahn School of Medicine at Mount Sinai, New York, NY 10029, USA; ene.kahn@mssm.edu
[6] Department of Psychiatry and Biobehavioral Medicine, Michigan State University, East Lansing, MI 48824, USA
* Correspondence: kthakkar@msu.edu

Exploratory Whole Brain Analysis

Methods

We conducted an exploratory whole brain analysis, examining the contrasts described in the manuscript (redirect versus fixation, no-step versus fixation, and redirect versus no-step) using uncorrected voxel thresholds $p < 0.001$. Statistical maps were masked to include voxels within grey matter and significant clusters were defined based on $p < 0.05$ FWE-corrected for multiple cluster-level comparisons.

Results

Significant within-group activation clusters for the examined contrasts are presented in **Table S1** and **Figure S1**. At a whole-brain level, in the redirect > no-step contrast, HC showed activation in a large network of frontal, parietal, and occipital regions. Of particular interest were regions involved in saccade planning and execution such as bilateral FEF, bilateral SEF extending into right ACC, and right IFC for redirect versus no-step trials. In SIB, similar regions as HC showed a difference but they showed qualitatively less activation with the exception of bilateral insula, left IFC and left inferior parietal. No differences between groups in the redirect versus no-step contrast reached significance.

Examination of within group effects in the redirect versus fixation and no-step versus fixation contrasts separately revealed similar patterns of activity in both groups but with qualitatively greater extent in the SIB group. The relatively reduced redirect versus no-step activation in SIB was driven by relatively more no-step activation that was more similar to redirect among SIB. Interestingly, in both no-step greater than fixation and redirect greater than fixation contrasts, group differences were seen in bilateral anterior cingulate with more activation in SIB.

Comparison of Sample Characteristics Between Experiment 1 and Experiment 2

Methods

In Experiment 1, we found significant differences in SSRT between HC and relatives, but in Experiment 2 the difference in TSRT between groups was marginal. In order to test whether differences between the two samples of unaffected relatives might explain these small differences across studies, we compared IQ, age, and social functioning as measured

by the Social Functioning Scale (SFS; Birchwood, Smith, Cochrane, Wetton, & Copestake, 1990) employment subscale score and total score using two-sample t-tests.

Results

Comparisons of relative groups showed no significant differences in age (t(32)=-1.93, p = 0.063), IQ (t(32) = -0.45, p = 0.656), SFS employment subscale (t(31)=1.23, p=0.229), or SFS total (t(31)=1.61, p = 0.118). The relatives in Experiment 1 were older (mean = 36.5 years, standard deviation = 11.01) than the siblings in Experiment 2 (mean = 31.09 years, standard deviation = 5.46), but this difference did not reach significance.

Calculation of Bayes Factors for SSRT and TSRT in Experiment 1 and Experiment 2 Respectively

Methods

Given our sample sizes it is worth examining the likelihood of the null and alternative hypotheses given our sample and effect sizes. Therefore, we calculated Bayes factors for the t-test comparing SSRT between HC and REL groups in Experiment 1 and the t-test comparing TSRT between HC and SIB groups in Experiment 2. We used the online calculator provided online (http://pcl.missouri.edu/bf-two-sample) that implements the procedure from Rouder et al. (2009). We used an r scale value of 1 as suggested in the manuscript and report the JZS Bayes Factor value.

Results

For the comparison of SSRT between HC and REL in Experiment 1 the scaled JZS Bayes Factor is 2.548. For the comparison of TSRT between HC and SIB in Experiment 2 the scaled JZS Bayes Factor is 1.115. These results suggest weak or anecdotal evidence supporting longer SSRT/TSRT in unaffected relatives (Jeffreys, 1961; Raftery, 1995; Wetzels et al., 2011).

Group Differences in the relationship between DCM parameters and TSRT

Methods

For each of the parameters that was associated with TSRT using the backwards elimination procedure for either group we ran follow up regression analyses that predicted TSRT for all participants in the neuroimaging analysis based on parameter values, group, and parameter by group interactions. This was done to examine whether group moderates the relationship between parameter values and TSRT. All models took the form TSRT~ group + param_values + group*param_values. Group was coded as 1 for siblings and -1 for healthy controls.

Results

Two of the parameters identified as showing significant relationships within either group showed significant results when investigated across groups. For the connection from FEF to caudate, group significantly predicted TSRT (p=0.002) with siblings showing longer TSRTs. The main effect of parameter values and the interaction between group and parameter values were not significant. For the connection from the rIFC to the SEF, more excitatory parameter values across both groups were associated with reduced TSRT (p=0.032); however, there were no effects of group or interaction with group. None of the other parameters showed significant relationships when considered across groups (see Table S5).

Supplementary Figure Legends

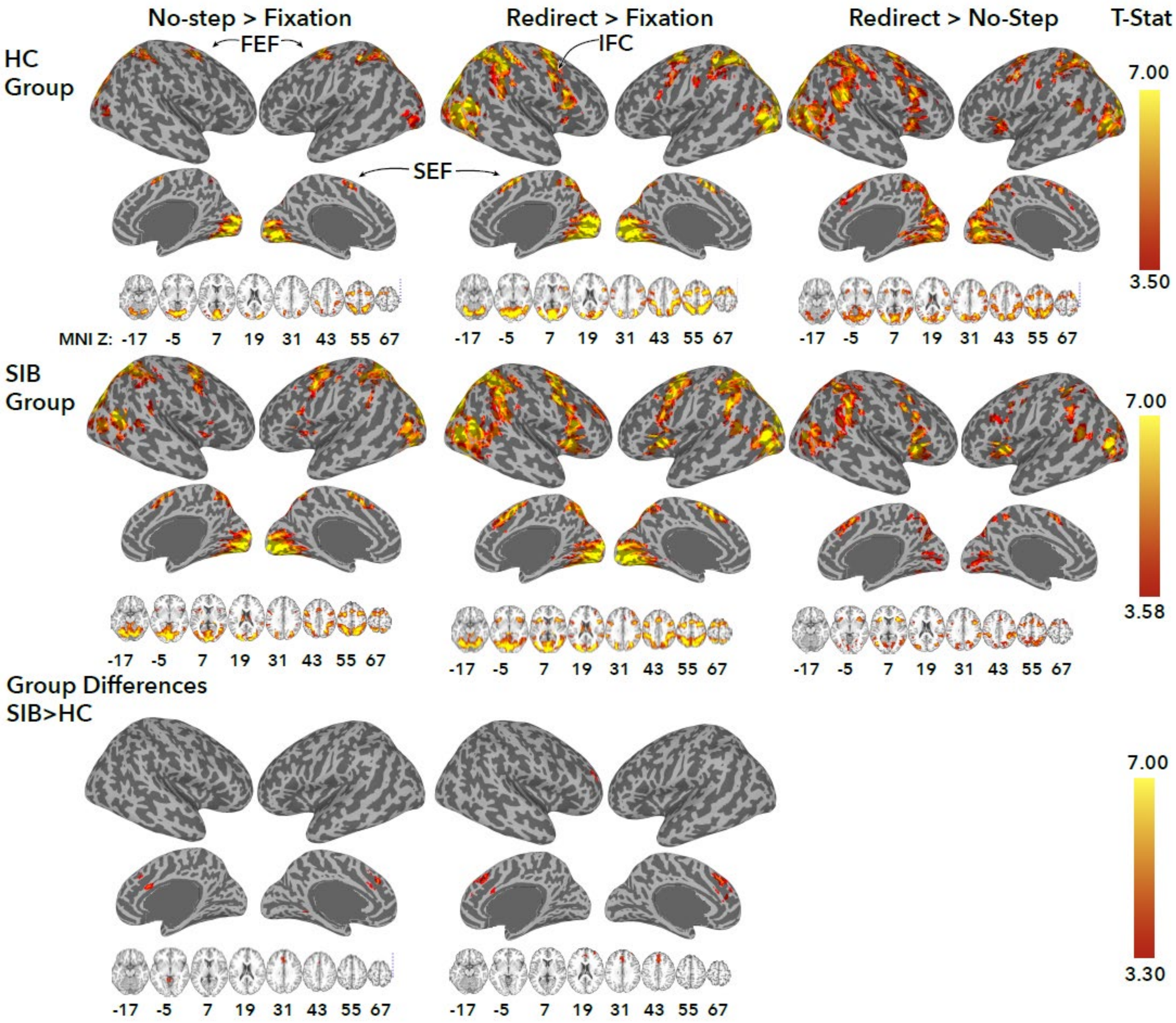

Figure S1. GLM results for healthy controls (top row), siblings (middle row), and their comparison (bottom row) for 3 contrasts: no-step greater than fixation (left column), redirect greater than fixation (middle column), and redirect greater than no-step (right column). No areas showed significant effects for the between group comparisons in the redirect greater than no-step condition. Minimum T-statistics were set to an uncorrected p = 0.001. Only clusters that had a FWE corrected p-value p< .05 using cluster-wise multiple corrections are visualized. For the group comparisons warm colors reflect the contrast of siblings greater than healthy controls. No regions were identified were healthy controls showed more activation than siblings. Slices show the same effects visualized at the labeled MNI Z axis slice number. Slices are visualized on a skull stripped MNI colin27 template using MRIcron, and surface images are displayed on an MNI surface in Afni's SUMA visualization tool.

Supplementary Tables

Table S1. GLM results.

Analysis	Cluster Size (Voxels)	Cluster wise P (FWE-corr)	Peak T-stat	Peak MNI Coordinates (mm) x	y	z	Region
			Controls: No-Step > Fixation				
	3362	0	12.92	-6	-78	-3	Left Lingual
			12.24	12	-93	6	Right Calcarine
			11.18	-21	-63	57	Left Superior Parietal
			9.87	18	-75	-9	Right Lingual
			9.15	21	-63	60	Right Superior Parietal
			9.01	-18	-78	-9	Left Lingual
			8.17	-9	-96	12	Left Superior Occipital
			7.88	-30	-57	51	Left Inferior Parietal
			7.87	-15	-90	0	Left Lingual
			7.83	-36	-48	51	Left Inferior Parietal
	228	0	9.02	24	-3	48	Right Precentral
			6.51	39	-3	48	Right Precentral
	273	0	9	-24	-3	54	Left Superior Frontal
			7.05	-45	-3	54	Left Precentral
	119	0.001	5.85	-3	3	57	Left Supplementary Motor Area
			5.78	6	6	60	Right Supplementary Motor Area
			5.67	-6	-3	66	Left Supplementary Motor Area
			Siblings: No-Step > Fixation				
	7076	0	14.82	-9	-96	12	Left Superior Occipital
			14.56	6	-81	-6	Right Lingual
			12.88	12	-90	0	Right Calcarine
			11.54	-3	-69	-3	Left Lingual
			10.73	12	-96	12	Right Cuneus
			10.57	24	-63	42	Right Superior Occipital
			10.42	-30	-57	-30	Left Cerebelum
			10.38	21	-69	51	Right Superior Parietal
			10.36	-15	-63	60	Left Precuneus
			9.86	-21	-78	-12	Left Lingual
	67	0.003	10.56	24	-30	6	Right Thalamus
			5.04	12	-18	15	Right Thalamus
	1360	0	8.6	30	0	54	Right Middle Frontal
			8.45	-27	-6	60	Left Superior Frontal
			8.1	-51	6	42	Left Precentral
			8.09	42	0	51	Right Precentral
			7.57	-6	-3	63	Left Supplementary Motor Area
			7.25	-45	-3	48	Left Precentral
			7.21	0	9	54	Left Supplementary Motor Area
			7.03	-30	0	66	Left Superior Frontal
			6.88	-24	-9	45	Left Precentral
			6.58	9	6	66	Right Supplementary Motor Area
	101	0	6.52	-18	-27	15	Left Thalamus
			6.43	-12	-21	15	Left Thalamus
			6.42	-21	-33	6	Left Thalamus
			4.29	-18	-6	21	Left Caudate
	98	0	5.99	-24	6	6	Left Putamen
			4.89	-42	3	3	Left Insula

		4.81	-36	18	3	Left Insula
		4.47	-54	15	-3	Left Superior Temporal Pole
		3.93	-51	9	6	Left Inferior Frontal Operculum
		3.91	-30	18	9	Left Insula
76	0.001	5.31	54	12	-15	Right Superior Temporal Pole
		4.76	45	12	0	Right Insula
		Controls: Redirect > Fixation				
7361	0	17.04	12	-90	6	Right Calcarine
		16.58	-3	-75	0	Left Lingual
		14.35	12	-60	54	Right Precuneus
		13.72	-21	-63	57	Left Superoir Parietal
		13.68	-18	-78	-9	Left Lingual
		13.51	-27	-75	21	Left Middle Occipital
		12.99	18	-69	57	Right Superior Parietal
		12.77	-9	-93	6	Left Calcarine
		12.29	18	-90	12	Right Cuneus
		11.94	48	-66	9	Right Middle Temporal
1810	0	11.99	-24	0	54	Left Superior Frontal
		11.78	36	3	57	Right Middle Frontal
		10.69	27	-3	51	Right Precentral
		9.08	51	9	21	Right Inferior Frontal Operculum
		8.98	-30	0	66	Left Superior Frontal
		8.79	-36	-3	51	Left Precentral
		8.52	0	0	66	Left Supplementary Motor Area
		7.94	42	-3	48	Right Precentral
		7.88	54	12	33	Right Precentral
		7.84	-3	6	54	Left Supplementary Motor Area
		Siblings: Redirect > Fixation				
9267	0	14.57	-9	-96	12	Left Superior Occipital
		14.08	-45	-72	12	Left Middle Occipital
		12.84	6	-84	-6	Right Lingual
		12.63	12	-90	0	Right Calcarine
		12.22	-30	-78	-9	Left Inferior Occipital
		11.9	-24	-84	30	Left Superior Occipital
		11.81	-3	-84	-6	Left Calcarine
		11.64	-33	-78	21	Left Middle Occipital
		11.48	27	-81	21	Right Superior Occipital
		11.48	-15	-66	63	Left Precuneus
2862	0	10.72	27	0	51	Right Precentral
		9.88	-30	-3	54	Left Middle Frontal
		9.4	-30	0	66	Left Superior Frontal
		9.06	-18	3	69	Left Superior Frontal
		8.86	-45	6	36	Left Precentral
		8.8	45	6	33	Right Precentral
		8.76	-45	-3	45	Left Precentral
		8.61	45	9	18	Right Inferior Frontal Operculum
		8.57	-30	18	9	Left Insula
		8.5	48	0	51	Right Precentral
158	0	6.07	39	36	33	Right Middle Frontal
		5.32	42	42	27	Right Middle Frontal
		5.05	36	42	21	Right Middle Frontal
		4.92	39	51	21	Right Middle Frontal
		Controls: Redirect > No-Step				

7011	0	10.07	27	-84	18	Right Middle Occipital
		9.64	24	-66	51	Right Superior Parietal
		8.93	-15	-75	39	Left Cuneus
		8.25	63	-42	24	Right Superior Temporal
		8.22	21	-66	33	Right Superior Occipital
		8.22	-30	-54	51	Left Inferior Parietal
		8.13	48	-69	0	Right Middle Temporal
		8.05	-15	-60	57	Left Precuneus
		7.93	-30	-42	51	Left Inferior Parietal
		7.92	-27	-81	33	Left Middle Occipital
1761	0	8.6	27	0	63	Right Superior Frontal
		7.59	-24	-9	57	Left Superior Frontal
		7.51	-54	3	42	Left Precentral
		7.23	30	-3	51	Right Precentral
		7.01	54	9	33	Right Precentral
		6.66	12	15	60	Right Supplementary Motor Area
		6.59	-33	-3	45	Left Precentral
		6.55	51	9	24	Right Inferior Frontal Operculum
		6.4	-21	0	54	Left Superior Frontal
		6.31	42	9	30	Right Inferior Frontal Operculum
66	0.005	5.85	-51	-48	12	Left Middle Temporal
		4.25	-66	-45	9	Left Middle Temporal
124	0	5.7	-33	21	6	Left Insula
		4.88	-27	21	-9	Left Insula
		4.64	-39	15	-6	Left Insula
43	0.032	5.05	-9	-24	12	Left Thalamus
		4.86	-12	-12	15	Left Thalamus
		4.25	-18	-30	15	Left Thalamus
		Siblings: Redirect > No-Step				
317	0	9.8	-30	18	9	Left Insula
		8.65	-42	12	3	Left Insula
		6.28	-45	3	36	Left Precentral
		5.53	-39	18	24	Left Inferior Frontal Triangularis
		4.69	-48	9	15	Left Inferior Frontal Operculum
		4.34	-45	3	24	Left Precentral
1129	0	9.72	36	18	3	Right Insula
		7.7	27	3	60	Right Superior Frontal
		7.35	42	3	33	Right Precentral
		7.05	27	-3	54	Right Superior Temporal
		6.9	45	9	21	Right Inferior Frontal Operculum
		6.49	30	12	57	Right Middle Frontal
		6.47	-30	0	57	Left Middle Frontal
		6.38	6	15	51	Right Supplementary Motor Area
		6.32	-15	6	63	Left Supplementary Motor Area
		6.21	-6	3	63	Left Supplementary Motor Area
2587	0	8.5	-45	-75	12	Left Middle Occipital
		8.27	-42	-69	6	Left Middle Occipital
		8.04	42	-72	15	Right Middle Occipital
		8.04	60	-39	36	Right Supramarginal
		7.22	9	-66	51	Right Precuneus
		6.99	30	-69	30	Right Middle Occipital
		6.89	54	-63	12	Right Middle Temporal
		6.85	36	-51	51	Right Inferior Parietal

		6.78	39	-45	42	Right Inferior Parietal
		6.5	60	-33	48	Right Supramarginal
234	0	6.83	-36	-39	45	Left Inferior Parietal
		5.34	-54	-36	36	Left Supramarginal
		4.77	-63	-39	30	Left Supramarginal
		4.7	-30	-48	54	Left Inferior Parietal
		4.2	-39	-48	45	Left Inferior Parietal
102	0	6.03	-48	-45	12	Left Middle Temporal
		4.85	-63	-57	9	Left Middle Temporal
		3.92	-60	-63	3	Left Middle Temporal
58	0.006	4.92	27	-60	-3	Right Fusiform
		4.56	33	-63	-12	Right Fusiform
		4.15	24	-75	-9	Right Fusiform
Siblings > Controls: No-Step > Fixation						
83	0.005	5.04	6	-42	-9	Vermis
		4.99	-6	-51	-6	Left Cerebellum
		4.31	0	-54	0	Vermis
		3.56	-15	-54	-12	Left Cerebellum
111	0.001	4.61	3	18	27	Right Anterior Cingulum
		4.55	-9	33	30	Left Anterior Cingulum
		3.97	0	24	36	Left Middle Cingulum
Siblings > Controls: Redirect > Fixation						
275	0	4.85	0	27	42	Left Superior Medial Frontal
		4.72	-3	33	33	Left Superior Medial Frontal
		4.02	-3	45	24	Left Superior Medial Frontal
		3.93	3	42	18	Right Anterior Cingulate
		3.9	0	18	42	Left Superior Medial Frontal
		3.76	0	15	30	Left Anterior Cingulate
		3.72	-9	39	15	Left Anterior Cingulate
		3.63	3	21	24	Right Anterior Cingulate
68	0.01	4.29	36	51	21	Right Middle Frontal
		4.04	30	48	39	Right Middle Frontal

Table S2. Control DCM parameters.

Connection type	Connection	BMA Probability	BMA parameter
Mean Effective Connectivity	From FEF to FEF	0.89	-0.08
Mean Effective Connectivity	From FEF to SEF	1.00	-0.25
Mean Effective Connectivity	From FEF to CD	0.00	0.00
Mean Effective Connectivity	From FEF to Thal	1.00	-0.07
Mean Effective Connectivity	From FEF to SC	0.00	0.00
Mean Effective Connectivity	From SEF to FEF	1.00	0.21
Mean Effective Connectivity	From SEF to SEF	1.00	-0.21
Mean Effective Connectivity	From SEF to CD	1.00	0.08
Mean Effective Connectivity	From SEF to Thal	0.00	0.00
Mean Effective Connectivity	From SEF to SC	1.00	-0.24
Mean Effective Connectivity	From SEF to IFC	0.00	0.00
Mean Effective Connectivity	From CD to CD	1.00	-0.61
Mean Effective Connectivity	From CD to Thal	1.00	0.08
Mean Effective Connectivity	From CD to SC	1.00	0.37
Mean Effective Connectivity	From Thal to FEF	1.00	0.43

Mean Effective Connectivity	From Thal to SEF	1.00	0.41
Mean Effective Connectivity	From Thal to Thal	1.00	-0.56
Mean Effective Connectivity	From Thal to IFC	1.00	0.29
Mean Effective Connectivity	From SC to CD	1.00	0.11
Mean Effective Connectivity	From SC to Thal	1.00	0.16
Mean Effective Connectivity	From SC to SC	1.00	-0.50
Mean Effective Connectivity	From IFC to SEF	1.00	0.11
Mean Effective Connectivity	From IFC to CD	1.00	-0.06
Mean Effective Connectivity	From IFC to Thal	0.00	0.00
Mean Effective Connectivity	From IFC to IFC	1.00	-0.37
Connection type	Connection	BMA Probability	BMA parameter
Modulation due to Redirect	From FEF to FEF	1.00	-1.41
Modulation due to Redirect	From FEF to SEF	0.68	0.26
Modulation due to Redirect	From FEF to CD	0.00	0.00
Modulation due to Redirect	From FEF to Thal	0.00	0.00
Modulation due to Redirect	From FEF to SC	0.00	0.00
Modulation due to Redirect	From SEF to FEF	0.00	0.00
Modulation due to Redirect	From SEF to SEF	0.86	-0.66
Modulation due to Redirect	From SEF to CD	0.94	0.38
Modulation due to Redirect	From SEF to Thal	0.00	0.00
Modulation due to Redirect	From SEF to SC	0.95	0.46
Modulation due to Redirect	From SEF to IFC	1.00	1.26
Modulation due to Redirect	From CD to CD	1.00	-1.88
Modulation due to Redirect	From CD to Thal	0.69	-0.34
Modulation due to Redirect	From CD to SC	0.00	0.00
Modulation due to Redirect	From Thal to FEF	0.87	-0.56
Modulation due to Redirect	From Thal to SEF	1.00	-1.15
Modulation due to Redirect	From Thal to Thal	1.00	-2.34
Modulation due to Redirect	From Thal to IFC	0.87	-0.53
Modulation due to Redirect	From SC to CD	0.00	0.00
Modulation due to Redirect	From SC to Thal	0.00	0.00
Modulation due to Redirect	From SC to SC	1.00	-1.56
Modulation due to Redirect	From IFC to SEF	1.00	1.12
Modulation due to Redirect	From IFC to CD	0.00	0.00
Modulation due to Redirect	From IFC to Thal	0.00	0.00
Modulation due to Redirect	From IFC to IFC	0.00	0.00

Note: The PEB and BMA model was not optimized over the C matrix, so C parameters are not estimated in the BMA.

Table S3. Sibling DCM parameters.

**Connection type**	**Connection**	**BMA Probability**	**BMA parameter**
Mean Effective Connectivity	From FEF to FEF	0.00	0.00
Mean Effective Connectivity	From FEF to SEF	1.00	-0.39
Mean Effective Connectivity	From FEF to CD	1.00	-0.09
Mean Effective Connectivity	From FEF to Thal	1.00	-0.06
Mean Effective Connectivity	From FEF to SC	1.00	-0.35
Mean Effective Connectivity	From SEF to FEF	1.00	0.10
Mean Effective Connectivity	From SEF to SEF	1.00	-0.57
Mean Effective Connectivity	From SEF to CD	1.00	-0.13
Mean Effective Connectivity	From SEF to Thal	0.00	0.00
Mean Effective Connectivity	From SEF to SC	1.00	0.07
Mean Effective Connectivity	From SEF to IFC	0.55	0.03

Mean Effective Connectivity	From CD to CD	1.00	-0.57
Mean Effective Connectivity	From CD to Thal	1.00	0.09
Mean Effective Connectivity	From CD to SC	1.00	0.14
Mean Effective Connectivity	From Thal to FEF	1.00	0.39
Mean Effective Connectivity	From Thal to SEF	1.00	0.52
Mean Effective Connectivity	From Thal to Thal	1.00	-0.82
Mean Effective Connectivity	From Thal to IFC	1.00	0.17
Mean Effective Connectivity	From SC to CD	1.00	0.23
Mean Effective Connectivity	From SC to Thal	0.56	0.03
Mean Effective Connectivity	From SC to SC	1.00	-0.62
Mean Effective Connectivity	From IFC to SEF	1.00	-0.13
Mean Effective Connectivity	From IFC to CD	0.00	0.00
Mean Effective Connectivity	From IFC to Thal	0.00	0.00
Mean Effective Connectivity	From IFC to IFC	1.00	-0.21
**Connection type**	**Connection**	**BMA Probability**	**BMA parameter**
Modulation due to Redirect	From FEF to FEF	1.00	-1.54
Modulation due to Redirect	From FEF to SEF	0.00	0.00
Modulation due to Redirect	From FEF to CD	0.00	0.00
Modulation due to Redirect	From FEF to Thal	0.00	0.00
Modulation due to Redirect	From FEF to SC	0.00	0.00
Modulation due to Redirect	From SEF to FEF	0.69	-0.23
Modulation due to Redirect	From SEF to SEF	1.00	-2.11
Modulation due to Redirect	From SEF to CD	1.00	0.56
Modulation due to Redirect	From SEF to Thal	1.00	0.77
Modulation due to Redirect	From SEF to SC	0.76	0.28
Modulation due to Redirect	From SEF to IFC	1.00	0.88
Modulation due to Redirect	From CD to CD	1.00	-2.97
Modulation due to Redirect	From CD to Thal	0.85	0.55
Modulation due to Redirect	From CD to SC	0.00	0.00
Modulation due to Redirect	From Thal to FEF	1.00	-0.67
Modulation due to Redirect	From Thal to SEF	0.00	0.00
Modulation due to Redirect	From Thal to Thal	1.00	-1.72
Modulation due to Redirect	From Thal to IFC	0.00	0.00
Modulation due to Redirect	From SC to CD	1.00	-0.96
Modulation due to Redirect	From SC to Thal	1.00	-0.49
Modulation due to Redirect	From SC to SC	0.83	-0.66
Modulation due to Redirect	From IFC to SEF	0.00	0.00
Modulation due to Redirect	From IFC to CD	0.00	0.00
Modulation due to Redirect	From IFC to Thal	0.87	-0.45
Modulation due to Redirect	From IFC to IFC	0.60	-0.29

Note: The PEB and BMA model was not optimized over the C matrix, so C parameters are not estimated in the BMA.

Table S4. Group DCM analysis.

**Connection Type**	**Connection**	**BMA Probability**	**BMA parameter**
Mean Effective Connectivity	From FEF to FEF	0.00	0.00
Mean Effective Connectivity	From FEF to SEF	1.00	-0.30
Mean Effective Connectivity	From FEF to CD	1.00	-0.06
Mean Effective Connectivity	From FEF to Thal	1.00	-0.08
Mean Effective Connectivity	From FEF to SC	1.00	-0.21
Mean Effective Connectivity	From SEF to FEF	1.00	0.10
Mean Effective Connectivity	From SEF to SEF	1.00	-0.25
Mean Effective Connectivity	From SEF to CD	0.00	0.00
Mean Effective Connectivity	From SEF to Thal	0.00	0.00
Mean Effective Connectivity	From SEF to SC	1.00	-0.08
Mean Effective Connectivity	From SEF to IFC	0.00	0.00
Mean Effective Connectivity	From CD to CD	1.00	-0.62
Mean Effective Connectivity	From CD to Thal	1.00	0.07
Mean Effective Connectivity	From CD to SC	1.00	0.25
Mean Effective Connectivity	From Thal to FEF	1.00	0.40
Mean Effective Connectivity	From Thal to SEF	1.00	0.46
Mean Effective Connectivity	From Thal to Thal	1.00	-0.63
Mean Effective Connectivity	From Thal to IFC	1.00	0.23
Mean Effective Connectivity	From SC to CD	1.00	0.13
Mean Effective Connectivity	From SC to Thal	1.00	0.11
Mean Effective Connectivity	From SC to SC	1.00	-0.55
Mean Effective Connectivity	From IFC to SEF	0.00	0.00
Mean Effective Connectivity	From IFC to CD	0.00	0.00
Mean Effective Connectivity	From IFC to Thal	0.00	0.00
Mean Effective Connectivity	From IFC to IFC	1.00	-0.24
Connection Type	Connection	BMA Probability	BMA parameter
Mean Modulation	From FEF to FEF	1.00	-1.41
Mean Modulation	From FEF to SEF	0.00	0.00
Mean Modulation	From FEF to CD	0.00	0.00
Mean Modulation	From FEF to Thal	0.54	-0.12
Mean Modulation	From FEF to SC	0.00	0.00
Mean Modulation	From SEF to FEF	0.00	0.00
Mean Modulation	From SEF to SEF	1.00	-1.61
Mean Modulation	From SEF to CD	1.00	0.48
Mean Modulation	From SEF to Thal	1.00	0.32
Mean Modulation	From SEF to SC	1.00	0.47
Mean Modulation	From SEF to IFC	1.00	1.13
Mean Modulation	From CD to CD	1.00	-2.17
Mean Modulation	From CD to Thal	0.00	0.00
Mean Modulation	From CD to SC	0.00	0.00
Mean Modulation	From Thal to FEF	1.00	-0.56
Mean Modulation	From Thal to SEF	1.00	-0.73
Mean Modulation	From Thal to Thal	1.00	-1.70
Mean Modulation	From Thal to IFC	1.00	-0.54
Mean Modulation	From SC to CD	1.00	-0.48
Mean Modulation	From SC to Thal	0.00	0.00
Mean Modulation	From SC to SC	1.00	-0.88
Mean Modulation	From IFC to SEF	1.00	0.57
Mean Modulation	From IFC to CD	0.00	0.00
Mean Modulation	From IFC to Thal	0.00	0.00

Mean Modulation	From IFC to IFC	0.00	0.00
Connection Type	Connection	BMA Probability	BMA parameter
Group Differences in Mean Connectivity	From FEF to FEF	1.00	0.07
Group Differences in Mean Connectivity	From FEF to SEF	1.00	-0.06
Group Differences in Mean Connectivity	From FEF to CD	1.00	-0.04
Group Differences in Mean Connectivity	From FEF to Thal	0.00	0.00
Group Differences in Mean Connectivity	From FEF to SC	1.00	-0.16
Group Differences in Mean Connectivity	From SEF to FEF	0.00	0.00
Group Differences in Mean Connectivity	From SEF to SEF	1.00	-0.15
Group Differences in Mean Connectivity	From SEF to CD	1.00	-0.07
Group Differences in Mean Connectivity	From SEF to Thal	0.53	0.02
Group Differences in Mean Connectivity	From SEF to SC	1.00	0.15
Group Differences in Mean Connectivity	From SEF to IFC	0.00	0.00
Group Differences in Mean Connectivity	From CD to CD	0.00	0.00
Group Differences in Mean Connectivity	From CD to Thal	0.00	0.00
Group Differences in Mean Connectivity	From CD to SC	1.00	-0.10
Group Differences in Mean Connectivity	From Thal to FEF	0.00	0.00
Group Differences in Mean Connectivity	From Thal to SEF	0.00	0.00
Group Differences in Mean Connectivity	From Thal to Thal	1.00	-0.08
Group Differences in Mean Connectivity	From Thal to IFC	0.57	-0.04
Group Differences in Mean Connectivity	From SC to CD	0.00	0.00
Group Differences in Mean Connectivity	From SC to Thal	0.64	-0.03
Group Differences in Mean Connectivity	From SC to SC	1.00	-0.08
Group Differences in Mean Connectivity	From IFC to SEF	1.00	-0.11
Group Differences in Mean Connectivity	From IFC to CD	0.00	0.00
Group Differences in Mean Connectivity	From IFC to Thal	0.51	-0.02
Group Differences in Mean Connectivity	From IFC to IFC	1.00	0.13
Connection Type	Connection	BMA Probability	BMA parameter
Group Differences in Modulation	From FEF to FEF	0.00	0.00
Group Differences in Modulation	From FEF to SEF	0.00	0.00
Group Differences in Modulation	From FEF to CD	0.00	0.00
Group Differences in Modulation	From FEF to Thal	0.00	0.00
Group Differences in Modulation	From FEF to SC	0.00	0.00
Group Differences in Modulation	From SEF to FEF	1.00	-0.31
Group Differences in Modulation	From SEF to SEF	0.62	-0.29
Group Differences in Modulation	From SEF to CD	0.00	0.00
Group Differences in Modulation	From SEF to Thal	0.64	0.16
Group Differences in Modulation	From SEF to SC	0.00	0.00
Group Differences in Modulation	From SEF to IFC	0.00	0.00
Group Differences in Modulation	From CD to CD	0.00	0.00
Group Differences in Modulation	From CD to Thal	0.69	0.31
Group Differences in Modulation	From CD to SC	0.00	0.00
Group Differences in Modulation	From Thal to FEF	0.00	0.00
Group Differences in Modulation	From Thal to SEF	1.00	0.69
Group Differences in Modulation	From Thal to Thal	0.00	0.00
Group Differences in Modulation	From Thal to IFC	0.00	0.00
Group Differences in Modulation	From SC to CD	1.00	-0.30
Group Differences in Modulation	From SC to Thal	1.00	-0.35
Group Differences in Modulation	From SC to SC	1.00	0.58
Group Differences in Modulation	From IFC to SEF	1.00	-0.58
Group Differences in Modulation	From IFC to CD	0.00	0.00
Group Differences in Modulation	From IFC to Thal	0.00	0.00

Group Differences in Modulation	From IFC to IFC	0.00	0.00

Table S5. Regressions examining the relationship between DCM parameters and TSRT within both groups.

	**Intercept**		**Group Main Effect**		**Parameter Main Effect**		Group*Param Interaction		Model Fit		
**Parameter**	**Est.**	**P**	**Est.**	**P**	**Est.**	P	Est.	P	F(3,39)	P	Adj. R^2
FEF to FEF	138.39	**<0.001**	-10.31	0.589	299.96	0.285	354.85	0.208	1.69	0.186	0.05
FEF to SEF	168.26	**<0.001**	0.40	0.960	26.26	0.217	-28.67	0.182	1.96	0.136	0.06
FEF to CD	179.50	**<0.001**	26.51	**0.002**	154.93	0.518	265.25	0.270	3.73	**0.019**	0.16
SEF to CD	171.91	**<0.001**	27.02	0.180	169.84	0.348	58.33	0.746	1.44	0.246	0.03
SEF to SC	163.26	**<0.001**	-0.61	0.971	67.65	0.688	25.19	0.881	1.21	0.318	0.01
CD to SC	150.66	**<0.001**	17.47	0.222	32.11	0.458	-22.05	0.610	1.33	0.278	0.02
Thal to Thal	174.71	0.511	-185.61	0.485	76.38	0.824	-295.75	0.393	1.60	0.206	0.04
rIFC to SEF	127.78	**<0.001**	-32.57	0.093	-290.45	**0.032**	-232.49	0.082	3.12	**0.037**	0.13
rIFC to rIFC	162.57	**0.003**	-86.86	0.099	-116.94	0.588	-381.88	0.083	2.33	0.089	0.09
Modulation: Thalamus to SEF	160.39	**<0.001**	8.55	0.096	0.95	0.827	2.28	0.601	1.20	0.322	0.01

References

Birchwood, M., Smith, J., Cochrane, R., Wetton, S., & Copestake, S. (1990). The social functioning scale the development and validation of a new scale of social adjustment for use in family intervention programmes with schizophrenic patients. *The British Journal of Psychiatry, 157*(6), 853-859.

Jeffreys, H. (1961). Theory of probability (3rd edt.) oxford university press. *MR0187257, 432*.

Raftery, A. E. (1995). Bayesian model selection in social research. *Sociological methodology*, 111-163.

Rouder, J. N., Speckman, P. L., Sun, D., Morey, R. D., & Iverson, G. (2009). Bayesian t tests for accepting and rejecting the null hypothesis. *Psychonomic bulletin & review, 16*(2), 225-237.

Wetzels, R., Matzke, D., Lee, M. D., Rouder, J. N., Iverson, G. J., & Wagenmakers, E.-J. (2011). Statistical evidence in experimental psychology: An empirical comparison using 855 t tests. *Perspectives on Psychological Science, 6*(3), 291-298.
